# Engineering Interfacial Donor–Acceptor Molecular Cocrystals

**DOI:** 10.1021/acs.jpclett.5c03865

**Published:** 2026-01-29

**Authors:** Nikolai Severin, Neda Todorova, Tomáš Neveselý, Megan Davis, Martin Presselt, Filippo Giovanni Fabozzi, Stefan Hecht

**Affiliations:** † Department of Physics, 234383 Humboldt-Universität zu Berlin, 12489 Berlin, Germany; ‡ Center for the Science of Materials Berlin, 234383 Humboldt-Universität zu Berlin, 12489 Berlin, Germany; § Department of Chemistry, 234383 Humboldt-Universität zu Berlin, 12489 Berlin, Germany; ∥ Department of Mechanical Engineering, University of Colorado Boulder, Boulder, Colorado 80309, United States; ⊥ Institute of Physical Chemistry, Friedrich Schiller University Jena, 07743 Jena, Germany; # Leibniz Institute of Photonic Technology (IPHT), 07745 Jena, Germany; ∇ Sciclus GmbH & Co. KG, 07745 Jena, Germany; ○ Center for Energy and Environmental Chemistry Jena (CEEC Jena) Friedrich-Schiller University Jena, 07743 Jena, Germany

## Abstract

Y6 is a high-performance non-fullerene acceptor widely used in organic solar cells. The assembly behavior of Y6 at interfaces, however, remains mostly unknown and respective in-depth investigations are missing. Here, we use room temperature scanning tunneling microscopy to investigate the self-assembly of Y6 at the solid–liquid interface. We show how coadsorption of polycyclic aromatic hydrocarbon donor molecules leads to the formation of a series of donor–acceptor interfacial cocrystals with tailored molecular structure and order on a graphite surface. The gained understanding of the intricate interplay of the underlying intermolecular interactions should facilitate the engineering of multicomponent interfacial nanostructures and help to potentially improve charge separation and transport in organic (opto)­electronic devices.

## Graphical abstract

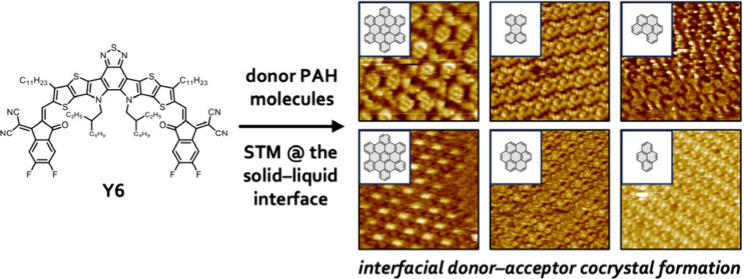

In recent years, non-fullerene acceptors (NFAs) have pushed the development of organic photovoltaics (OPVs) with record power conversion efficiencies (PCEs) reaching ∼20%.^1–4^ Unlike traditional fullerenes, NFAs possess distinct advantages such as tunable bandgap, tunable energy levels, and optimal control over the morphology of crystalline phases.^5^ Since its first discovery and implementation as active material in organic solar cells (OSCs), the NFA Y6 has demonstrated remarkable properties, reaching PCEs up to 18%.^6,7^ Structurally, Y6 exhibits an alternating donor–acceptor oligomeric structure (A^1^–D–A^2^–D–A^1^) in which two terminal dicyanomethylenedifluoroindanone acceptor (A^1^) moieties are connected through a central dithienopyrrole–benzothiadiazole donor–acceptor–donor (D–A^2^–D) core (Figure 1). The cyano groups and fluorine atoms boost the electron-withdrawing strength of Y6’s termini, while the long alkyl chains pending from the central π-conjugated core enhance its solubility in common organic solvents.

**Figure 1:**
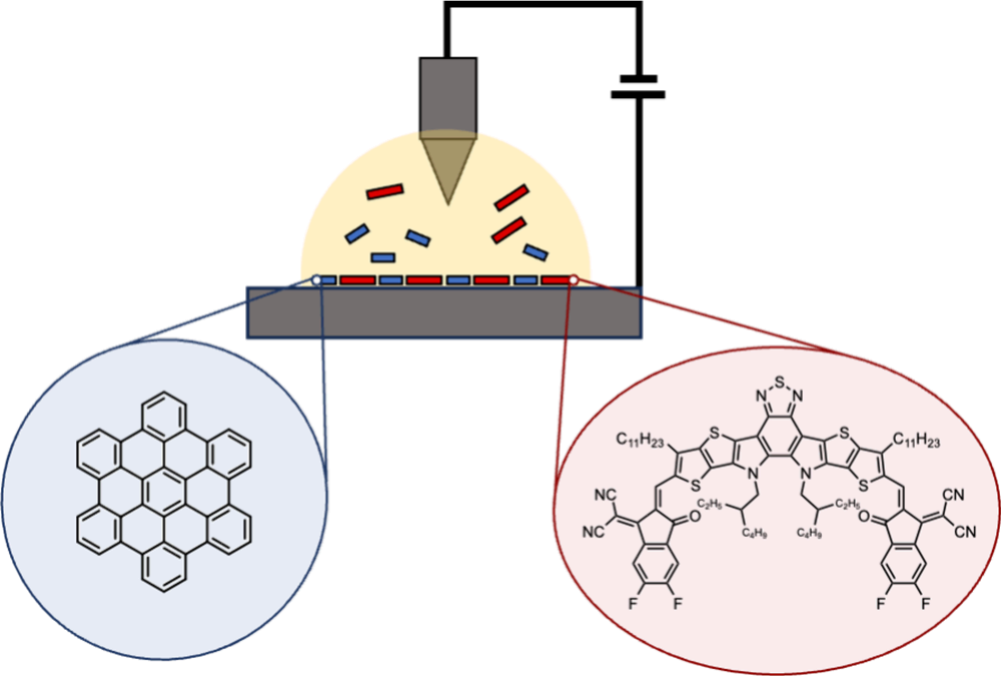
Interfacial cocrystal formation between molecular donor (blue) and acceptor (red) components on a semiconducting surface probed by STM at the solid–liquid interface. Hexabenzocoronene (HBC) serves here as the donor component (structure highlighted in the blue circle), while Y6 serves as the acceptor component (structure highlighted in the red circle).

In order to increase the PCE of OSCs, the NFA Y6 is typically blended with conjugated donor polymers.^8^ These D–A systems allow an enhanced electron mobility throughout the active layer, confining excitons within a diffusion length of a dissociating interface, thereby minimizing exciton recombination.^9,10^ Yet, understanding and controlling Y6 interfacial crystallization behavior as well as preventing phase separation at the interface remain key challenges for the design of D–A systems.^11^ In order to precisely engineer D–A cocrystals, it is critical to investigate the nanoscale assembly of Y6 with discrete donor molecules.^12,13^ Polycyclic aromatic hydrocarbons (PAHs) constitute an attractive class of such donor molecules^14–17^ and therefore exploring their interfacial cocrystallization behavior with Y6 holds great promise for the control over the active-layer morphology, potentially leading to increased electron/hole mobilities at the interface.^11,18–20^

Typically, electron diffraction techniques as well as UV–vis and vibrational spectroscopy are employed to gain information concerning the morphology of thin films;^21–23^ however, obtaining structural information at the nanoscale with molecular resolution remains challenging in the field. In this context, scanning tunneling microscopy (STM) at the solid–liquid interface has proven to be a powerful method for characterizing self-assembled molecular networks (SAMNs) with unprecedented lateral resolution.^24–26^

In this work, we investigate the crystallization behavior of Y6 in the absence and presence of PAH molecules on highly oriented pyrolytic graphite (HOPG) by using room temperature STM (Figure 1). We find that the combination of Y6 acceptor with PAH donor molecules leads to various novel two-dimensional D–A cocrystals at the solid–liquid interface. Our results illustrate how fine-tuning intermolecular interactions between different molecular building blocks at the interface facilitates the design of multicomponent active layers for (opto)­electronics devices.

A droplet of a Y6 solution (2 × 10^–5^ M in phenyloctane) was cast on a freshly cleaved HOPG surface and the resulting sample was measured by STM at the solid–liquid interface. Phenyloctane (1-PO) was used as STM solvent as it solely contributes with van der Waals interactions and π–π stacking, without inducing any strong directional bond that might influence the self-assembly of Y6. STM images revealed the successful formation of extended SAMNs spread across the entire HOPG surface (see Figure S1a in the Supporting Information). Interestingly, probing different areas revealed the formation of two polymorphs under the conditions mentioned above (Figure 2).

**Figure 2:**
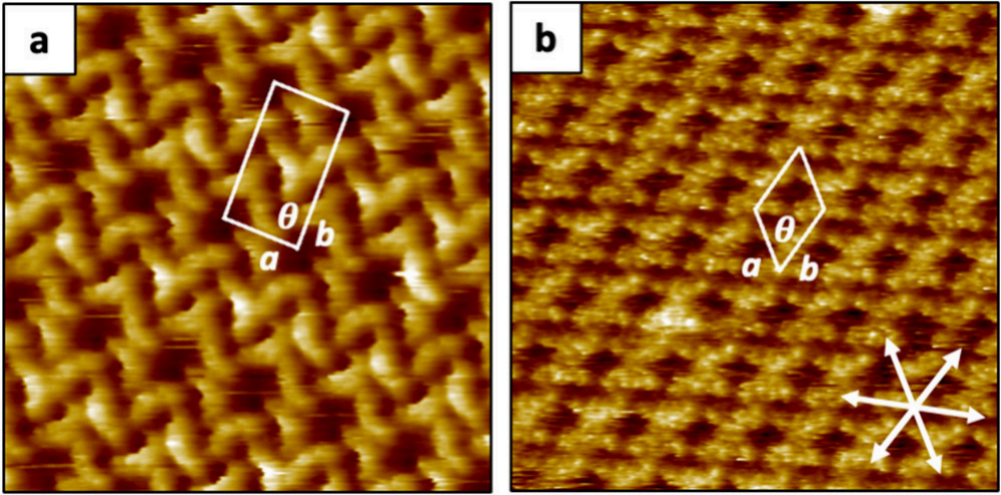
HR-STM images of SAMNs composed of Y6 molecules acquired at the solid–liquid interface (HOPG/1-PO): a) First polymorph P1_Y6_ consisting of five Y6 molecules per unit cell area (scanned area: 20 nm × 20 nm; *I* = 43 pA V *=* 1.35 V). b) Second polymorph P2_Y6_ consisting of two Y6 molecules per unit cell area (scanned area: 20 nm × 20 nm; *I* = 43 pA V *=* 1.35 V).

The first polymorph (P1_Y6_) represents the most commonly observed, composed by five Y6 molecules per unit cell (Figure 2a) with a surface packing density of ∼0.23 molecules·nm^–2^. FFT analysis revealed lattice parameters of *a* = 3.4 ± 0.1 nm, *b* = 6.0 ± 0.2 nm, and θ = 86 ± 3° (see Figure S1b in the Supporting Information). To better understand how Y6 organizes and orients within the supramolecular structure, truncated Y6 molecules were simulated by DFT calculations, scaled, and superimposed on the HR-STM images (see Figure S2a in the Supporting Information). Although the assembly in P1_Y6_ may appear fully periodic at first glance, detailed analysis of the HR-STM images revealed that some of the Y6 molecules randomly adopt one of two mirrored conformations on the basal plane of the HOPG surface (Figure 2a and Figure S3 in the Supporting Information). Moreover, we found that one Y6 molecule out of four is reproducibly characterized by brighter STM contrast features, associated with a change in apparent height, probably due to the out-of-plane bending of the long alkyl chains (see Figure S4 in the Supporting Information).^27^

The second polymorph (P2_Y6_) was rarely visualized under these STM conditions at the solid/liquid interface (HOPG/1-PO). It consists of two molecules per unit cell, giving a surface packing density of ∼0.27 molecules·nm^–2^. Y6 molecules arrange here in a dimer-like supramolecular structure with a star-shaped geometry (Figure 2b). HR-STM revealed a unit cell with lattice parameters *a* = *b* = 2.9 ± 0.1 nm. and θ = 61 ± 1°. Similarly, DFT simulated Y6 molecules were scaled and manually placed on top of the HR-STM image for the reader’s clarity (see Figure S2b in the Supporting Information).

Systematic changes of the environmental conditions, such as variation of the applied STM tip bias, to study the stability and responsiveness of Y6 SAMNs were also investigated. Changing of the current setpoint and the STM tip bias, i.e., moving the STM tip along the *z* direction normal to the surface, did not lead to any visualization of additional upper (or lower) molecular layer, implying that no ordered bilayer was formed (see Figures S1 and S2 in the Supporting Information). Furthermore, change in the orientation of the applied electric field, i.e., going from positive to negative STM tip bias, did not lead to any reorganization of the supramolecular organization, suggesting that Y6 forms a rather stable 2D SAMN (see Figure S5 in the Supporting Information).

To gain more insight into the interfacial crystallization behavior, we systematically varied the Y6 concentration. Interestingly, stable 2D SAMNs were visualized on HOPG at the solid–liquid interface only over a rather narrow concentration range (Figure 3). Specifically, when a droplet of a dilute Y6 solution with a concentration lower than 9.5 × 10^–6^ M was cast on a freshly cleaved HOPG surface, no stable assembly was observed by STM (Figure 3a). Increasing the concentration of the Y6 solution up to 4.8 × 10^–5^ M led to the successful formation of the previously observed supramolecular structures P1_Y6_ and P2_Y6_ (Figure 3b). Further increasing the concentration resulted in the formation and visualization of exclusively amorphous material (Figure 3c). Moreover, please note that regardless of the concentration of the Y6 solutions, no SAMN could be ever observed by STM at the solid–air interface (for further experimental details see the Materials and Methods).

**Figure 3:**
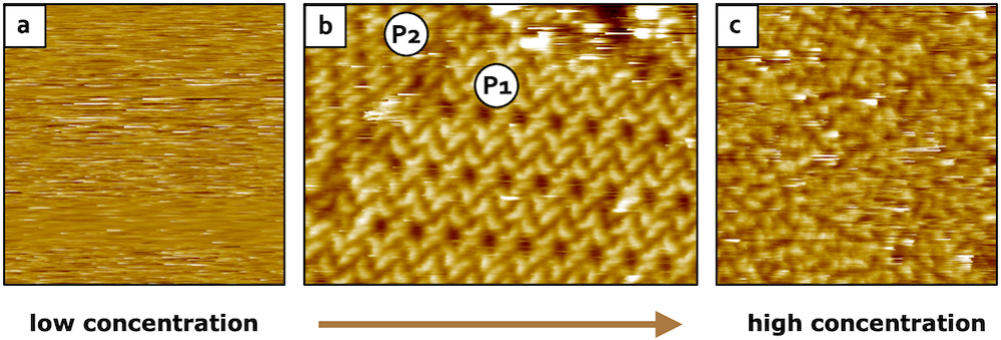
Variation of the Y6 concentration followed by STM at the solid–liquid interface (HOPG/1-PO): a) STM image obtained upon deposition of a dilute solution of Y6 (<9.5 × 10^–6^ M) on HOPG showing no assembly formation (scanned area: 30 nm × 30 nm; *I* = 49 pA V *=* 1.35 V). b) Increasing the concentration of the Y6 solution (4.8 × 10^–5^ M) led to the formation and visualization of stable SAMNs on HOPG (scanned area: 39 nm × 27 nm; *I* = 49 pA V *=* 1.35 V). c) Increasing the concentrations even further (>4.8 × 10^–5^ M) led to the formation and visualization of exclusively amorphous material (scanned area: 30 nm × 30 nm; *I* = 49 pA V *=* 1.05 V).

Since the solvent plays a major role in the formation and stabilization of 2D SAMNs,^28,29^ alternative solvents such as octanoic acid (OA) or 1,2,4-trichlorobenzene (TCB) were also investigated. Deposition of a Y6 solution in OA (2 × 10^–5^ M) on a freshly cleaved HOPG surface readily led to the formation of a stable SAMN (see Figure S6a in the Supporting Information). FFT analysis of the obtained HR-STM images revealed a distorted hexagonal unit cell with lattice parameters *a* = 2.9 ± 0.3 nm, *b* = 3.2 ± 0.1 nm, and θ = 63 ± 2° resembling the unit cell values of the second polymorph P2_Y6_ observed in 1-PO (Figure 2b). Similarly, deposition of a Y6 solution in TCB (2 × 10^–5^ M) on a freshly cleaved HOPG surface revealed the same dimer-like supramolecular structure (see Figure S6b in the Supporting Information) with lattice parameters *a* = *b* = 2.6 ± 0.2 nm and θ = 61°. While for 1-PO the first polymorph P1_Y6_ was almost exclusively found, in the case of OA and TCB apparently only the second polymorph P2_Y6_ was present on the HOPG surface.

As the main polymorph of Y6 at the HOPG/1-PO interface presents defined nanopores (voids visible in the STM image of Figure 2a), PAHs with disk-like shapes such as hexabenzocoronene (HBC) were codeposited with Y6 onto the HOPG surface. For this purpose, a droplet of a mixture of Y6 and HBC in 1-PO (Y6 = 2 × 10^–5^ M, HBC = saturated solution) was cast on a freshly cleaved HOPG surface and STM images were acquired at the solid–liquid interface (for further experimental details, see the Materials and Methods). Rather than revealing discrete host–guest complexes,^30^ to our surprise STM imaging showed complete reorganization of the 2D SAMN, resulting in the formation of extended interfacial cocrystals (see Figure S7 in the Supporting Information). Scanning over many different areas revealed the formation of two different polymorphs at the HOPG/1-PO interface (Figure 4). The predominant polymorph P1_Y6:HBC_ is characterized by a unit cell with lattice parameters of *a* = 3.9 ± 0.3 nm, *b* = 5.0 ± 0.2 nm, and θ = 44 ± 6° (Figure 4a). Here, alternating Y6 and HBC molecules are organized in quasi 1D rows, with the electron deficient sites of Y6 closely interacting with the HBCs’ outer π-cores. DFT simulated structures of Y6 and HBC are scaled and superimposed on the STM image for a better understanding of their orientation within the molecular coassembly (see Figure S8 in the Supporting Information). The second, less abundant polymorph P2_Y6:HBC_ exhibits unit cell parameters of *a* = 6.0 ± 0.7 nm, *b* = 5.3 ± 0.1 nm, and θ = 55 ± 2° (Figure 4b). In this case, obtaining submolecular resolution with STM under these conditions has been found to be particularly challenging. Cleaning of the STM tip with strong bias pulsing revealed that the bright features in the STM images are composed by two HBC molecules, surrounded by Y6 assemblies (see Figure S9 in the Supporting Information).

**Figure 4:**
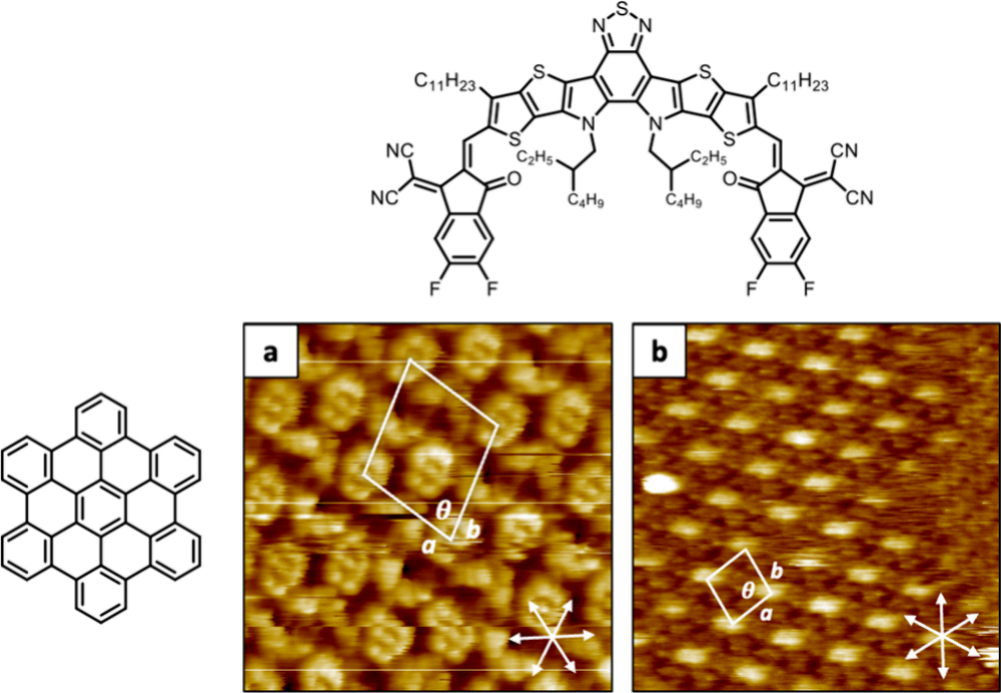
HR-STM images of Y6:HBC cocrystals formed at the solid–liquid interface (HOPG/1-PO): a) First predominant polymorph P1_Y6:HBC_ composed of alternating Y6 and HBC molecules organized in repeating 1D rows (scanned area: 10 nm × 10 nm; *I* = 79 pA V *=* 0.9 V). b) Second polymorph P2_Y6:HBC_ with two HBC molecules (bright contrast features) surrounded by Y6 molecules (scanned area: 30 nm × 30 nm; *I* = 49 pA V *=* 1.35 mV).

Also here, in order to understand whether and how the solvent affects the cocrystallization process, OA was employed for STM measurements. While keeping the concentration constant, a droplet of a mixed solution of Y6 and HBC was cast on a freshly cleaved HOPG surface, and STM images were obtained at the solid–liquid interface (HOPG/OA). While interfacial cocrystal formation was readily visible, no variation in the final supramolecular organization was observed, with both molecular components assembling in the same way as observed in Figure 4a (see Figure S10 in the Supporting Information), suggesting that the solvent does not play a major role in the cocrystallization process.

Since the formation of Y6:HBC cocrystals has proven to be readily accessible and highly reproducible, other PAH systems were tested for the interfacial cocrystallization process. Specifically, the codeposition of Y6 with perylene, pyrene, coronene, and benzo­[*ghi*]­perylene was investigated. A droplet of a mixed solution of Y6 and perylene in 1-PO (Y6 = 2 × 10^–5^ M, perylene = saturated solution) was cast on a freshly cleaved HOPG substrate and STM images at the solid–liquid interface (HOPG/1-PO) were obtained. HR-STM promptly revealed the formation of Y6:perylene cocrystals characterized by a unit cell with lattice parameters of *a* = 3.0 ± 0.1 nm, *b* = 3.4 ± 0.2 nm, and θ = 59 ± 1° (Figure 5a). Simulated structures of Y6 and perylene molecules were scaled and superimposed on the STM image for clarity (see Figure S11 in the Supporting Information). Differently from the Y6:HBC cocrystals (Figure 4a), where the ratio between both components was 1:1, this time only one perylene per two Y6 molecules has been found. This is most probably associated with the limited space between two adjacent Y6 molecules, with the small PAH molecules occupying the interstitial sites between the Y6 dimers. The same above procedure was employed for investigating Y6:pyrene cocrystals, and also in this case, the formation of Y6:pyrene cocrystals was readily visible (Figure 5b). FFT analysis provided unit cell parameters of *a* = 2.1 ± 0.1 nm, *b* = 3.9 ± 0.1 nm, and θ = 62 ± 1°, whereas HR-STM imaging revealed a 1:1 stoichiometry of Y6 and pyrene (see Figure S12 in the Supporting Information). In both cases, Y6 adopts a dimer-like supramolecular structure similar to the second polymorph P2_Y6_ as observed in Figure 2b, however, in this coassembly the two pyrene molecules are tightly stacked.

**Figure 5:**
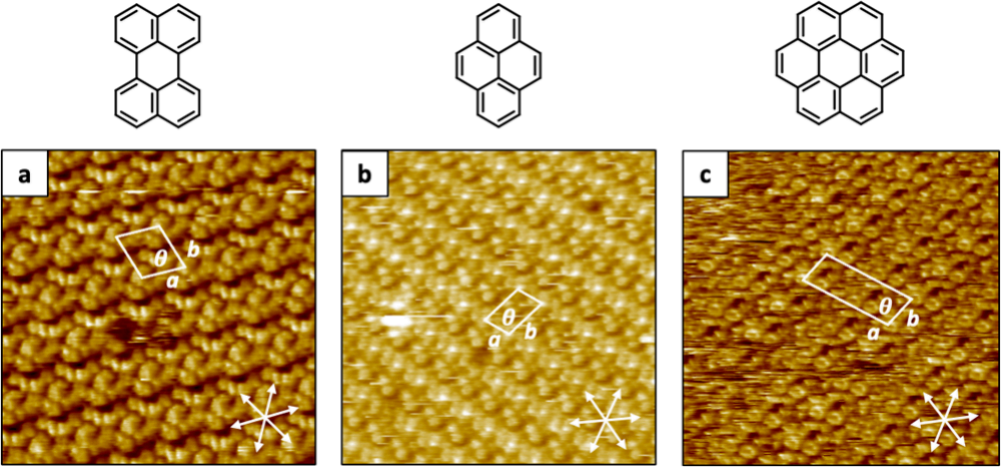
HR-STM images of various Y6:PAHs cocrystals formed at the solid–liquid interface (HOPG/1-PO): a) Y6:perylene cocrystal. (scanned area: 20 nm × 20 nm; *I* = 49 pA V *=* 1.35 V). b) Y6:pyrene cocrystal (scanned area: 20 nm × 20 nm; *I* = 49 pA V *=* 1.35 V). c) Y6:coronene cocrystal (scanned area: 25 nm × 25 nm; *I* = 49 pA V *=* 1.15 V).

To promote 2D cocrystal formation of Y6 with coronene and benzo­[*ghi*]­perylene, the PAH concentration was varied since deposition of their saturated solutions produced only amorphous material (see Figure S13 in the Supporting Information). Droplets of mixed solutions of Y6 with coronene and benzo­[*ghi*]­perylene, respectively (concentrations of each component were 2 × 10^–5^ M, giving rise to a 1:1 ratio), in 1-PO were deposited on freshly cleaved HOPG substrates, and the resulting assembly process was monitored by STM at the solid–liquid interface (HOPG/1-PO). In both cases, interfacial cocrystal formation was readily visible by STM (Figure 5c and see Figure S14 in the Supporting Information). FFT analysis of the STM images revealed unit cell parameters *a* = 2.91 ± 0.03 nm, *b* = 2.8 ± 0.2 nm, and θ = 86° as well as *a* = 6.4 ± 0.4 nm, *b* = 3.0 ± 0.2 nm, and θ = 87 ± 2° for Y6:coronene and Y6:benzo­[*ghi*]­perylene cocrystals, respectively. While codeposition of Y6 with perylene, pyrene, and coronene revealed extended 2D cocrystal formation (Figures 5a–c), Y6:benzo­[*ghi*]­perylene cocrystals exhibited a less regular structure, likely due to the lower symmetry of benzo­[*ghi*]­perylene and reflecting a marked packing difference between the different PAHs with Y6 molecules (see Figure S14b in the Supporting Information).

Given the observed propensity of Y6 to cocrystallize with PAH systems on the HOPG surface, we hypothesize that their coassembly is driven either by charge transfer interactions or by a combination of strong π−π stacking and dipole–dipole interactions between the electron-poor Y6 and the electron-rich PAH systems.^31,32^ To test our hypothesis, DFT calculations were performed using Orca 6.1.0 ^33^ with ωB97x-3c composite method.^34^

All molecular structures were obtained through energy minimization carried out in a vacuum followed by frequency calculation to confirm that the minimum was obtained (see Figure S15 and Figure S16 in the Supporting Information). Only the truncated version of Y6 was herein considered due to the presence of the peripherical long alkyl chains which confer high molecular flexibility and result in numerous possible conformers (see Figure S15 in the Supporting Information). HOMO–LUMO calculations revealed rather similar HOMO levels for all of the investigated chemical species. Instead, Y6 reveals a very deep LUMO energy compared to PAH molecules (see Table S1 in the Supporting Information). This value is associated with the π-conjugated central donor–acceptor–donor core, which facilitates electron delocalization within the backbone. In contrast, the LUMO energies of the PAHs are relatively high, reflecting their intrinsic electron-donating character (see Table S1 in the Supporting Information). Additionally, simulations of adiabatic fundamental gaps between the ionization potential (IP) and the electron affinity (EA) revealed consistent results with previous calculations (see Table S2 in the Supporting Information). Since exergonic charge transfer requires a negative Δ*E* = IP_donor_ – EA_acceptor_ < 0, the obtained positive values suggest that thermodynamically charge transfer here is disfavored. While the vicinity of Y6 with the PAHs in the crystal lattice on the HOPG surface could result in orbital hybridization that possibly generate partially delocalized charge transfer states in the ground state,^35,36^ this scenario could not be proven computationally. It is also important to notice that during STM imaging a strong electric field at the STM junction is applied, contributing to a substantial variation of the molecular orbital energies.^37^

Because our DFT calculations do not support charge transfer interaction, we instead attributed the formation of ordered cocrystals to simpler dipole–dipole and π−π stacking interactions.^38,39^ Maps of the electrostatic potential of Y6 and HBC were computed in order to understand the electron distribution within the molecular structure and rationalize their organization on the HOPG surface (see Figure S17 in the Supporting Information). While for Y6 the electron density is mainly localized on the CN-groups of the dicyanomethylene moiety and the central benzothiadiazole core (blue color), HBC displays an electron-deficient region all around the π-core. Comparison with the experimental STM images indeed reveals that the electron-rich regions of Y6 are in close contact with the neighboring HBC’s electron-poor periphery (see Figures S8 and S17 in the Supporting Information). Furthermore, no bilayer cocrystal structure visualized by STM points toward an efficient in-plane dipole–dipole interaction rather than electrostatically unfavorable multilayering.

The formation of donor–acceptor cocrystals between PAH systems and Y6 was investigated by STM at the solid–liquid interface. Initial deposition of pure Y6 resulted in formation of extended SAMNs on HOPG. Environmental conditions, such as variation of the concentration, solvent, or manipulation of the applied bias, led to a deeper understanding of the interfacial assembly behavior of Y6. We found that Y6 molecules form stable and long-range SAMNs only within a narrow concentration range. Furthermore, deposition of mixed solutions of Y6 with PAH donor molecules, such as HBC, coronene, pyrene, perylene, and benzo­[*ghi*]­perylene, resulted in the unprecedented formation of extended D–A cocrystals at the HOPG/1-PO interface that were fully characterized by STM. While for Y6:HBC two polymorphs could be observed, for all the other PAHs only one structure was found. Data analysis of the experimental results combined with DFT calculations suggests strong π−π stacking and dipole–dipole interactions rather than a charge transfer process between the electron-acceptor Y6 and the electron-donor PAHs. Further DFT calculations that take into account molecule–substrate interactions as well as the presence of strong local electric fields are needed to fully understand the intermolecular interactions between D–A systems at the HOPG/liquid interface.

Our approach provides fundamental insights into the interfacial crystallization behavior of Y6 and, moreover, enables the rational engineering of interfacial cocrystals with suitable PAH donor molecules. By combining molecular design of the individual components with scanning probe techniques, we could demonstrate precise control over complex coassembly structures and the resulting electronic interactions at the nanoscale, thereby offering a powerful platform to tailor organic (opto)­electronic interfaces.

## Supplementary Material





## Notes

Materials and methods, experimental procedures and details, as well as additional STM images, DFT calculations, and coordinates (PDF)
Transparent Peer Review report available (PDF)
