# Supplementary material for: Engineering Interfacial Donor–Acceptor Molecular Cocrystals
Source: J Phys Chem Lett. 2026 Jan 29;17(7):1972–7. doi: 10.1021/acs.jpclett.5c03865 (PMC12927019; doi:10.1021/acs.jpclett.5c03865)
Supplement: Supplementary file 1 [file jz5c03865_si_001.pdf]

Supporting Information

Engineering Interfacial Donor–Acceptor Molecular Cocrystals

Nikolai Severin[a,b], Neda Todorova[b,c], Tomáš Neveselý[b,c], Megan Davis[d], Martin Presselt[e,f,g,h], Filippo Giovanni Fabozzi[b,c]*, and Stefan Hecht[b,c]*

[a]Department of Physics, Humboldt-Universität zu Berlin, 12489 Berlin, Germany
[b]Center for the Science of Materials Berlin, Humboldt-Universität zu Berlin, 12489 Berlin, Germany
[c]Department of Chemistry, Humboldt-Universität zu Berlin, 12489 Berlin, Germany
[d]Department of Mechanical Engineering, University of Colorado Boulder, Boulder, CO, 80309 USA
[e]Institute of Physical Chemistry, Friedrich Schiller University Jena, 07743 Jena, Germany
[f]Leibniz Institute of Photonic Technology (IPHT), 07745 Jena, Germany
[g]Sciclus GmbH & Co. KG, 07745 Jena, Germany
[h]Center for Energy and Environmental Chemistry Jena (CEEC Jena) Friedrich-Schiller University Jena, 07743 Jena, Germany

Correspondence to: filippo.giovanni.fabozzi@hu-berlin.de and sh@chemie.hu-berlin.de

Table of Contents

MATERIALS AND METHODS ---------- **S2**

SCANNING TUNNELLING MICROSCOPY (STM) CHARACTERIZATION ---------- **S3**

DFT CALCULATIONS ---------- **S10**

REFERENCES ---------- **S20**

Materials and Methods

The molecular precursors Y6 (1-Materials Inc), HBC, coronene, perylene, benzo[*ghi*]perylene, and pyrene were used without further purifications as received from the suppliers (BLD). The same applies to the STM solvents 1-PO (TCI), 1,2,4-thrichlorobenzene (TCI), and octanoic acid (Sigma–Aldrich). To prepare clean samples, the top–surface of HOPG substrates (Bruker, 12 mm x 12 mm x 2 mm, ZYB grade) was mechanically exfoliated using scotch tape. All the STM measurements were carried out at room temperature and in constant–current mode by using a NaioSTM (Nanosurf, Switzerland). Data analysis was carried out using SPIP (Image Metrology, A/S, Lyngby, DK). STM images that include the HOPG axes as inset were corrected for piezo drift by acquiring a graphite image directly after acquisition of an image with molecules. The correction factors were applied assuming the HOPG unit cell to have hexagonal geometry and lattice parameters of 0.246 nm. All the HR-STM images were cropped from a bigger image. **Figure 2a** was deconvoluted using the calibrated unit cell parameters determined in **Figure S1**. Surface packing density was calculated as $\rho_S = \frac{N}{A}$ [molecules·nm^{-2}], where N is the number of molecules per unit cell and A is the unit cell area. ρ_S was obtained by counting the number of molecules within a single experimentally determined unit cell in the STM image. Molecules that are partially overlapping the unit cell boundary were counted fractionally according to their area within the unit cell.

All the calculations performed in this study were done using ORCA version 6.1.0.[1,2] The computational scheme consists of standard approach of optimization with subsequent frequency calculation to confirm that the minimum was obtained. Optimization, frequency calculation and other properties were calculated using composite method wB97x-3C developed by the Grimme research group.[3,4] This method is a complete package consisting of range-separated hybrid DFT functional with specially tailored vDZP basis set and D4 dispersion correction. Due to its flexibility the Y6 molecule can exist in many different conformers and thus computational requirements would be drastically increased if the complete molecule was used.[5] All of the calculations regarding Y6 were done with the truncated version for which the long alkyl chains were substituted with methyl groups. Visualization of the orbitals and molecular structures was done using Avogadro 2 software.[6] Electrostatic potential maps were generated using Chemcraft (Chemcraft_b770bt_win64).[7] This software was also used to prepare or modify the input file and to extract the necessary data from the output files.

Sample preparation

Self-Assembly of Y6 on HOPG

Freshly cleaved HOPG was initially mounted in the scanning tunnelling microscope. A droplet of a Y6 solution in 1-phenyloctane (1-PO) was then cast on the HOPG surface and STM was measured at the solid-liquid interface. Similarly, for the measurements carried out at in dry conditions a droplet of Y6 in EtOH (∼3% DMSO) was cast on a freshly cleaved HOPG surface. After complete evaporation of the solvent from the HOPG surface at room temperature, STM was then measured at the solid-air interface.

Interfacial Cocrystals Formation of Y6:PAHs on HOPG

A solution of Y6 with PAH molecules was prepared by mixing 5 μL of Y6 ($2 \cdot 10^{-5}$ M in 1-PO) with 5 μL of PAH (for HBC and perylene = saturated solution, while for pyrene and benzo[*ghi*]perylene ∼$2 \cdot 10^{-5}$ M). A droplet of the mixed Y6:PAH solution was then deposited on freshly cleaved HOPG and STM was measured at the solid-liquid interface.

Scanning Tunnelling Microscopy (STM) Characterization

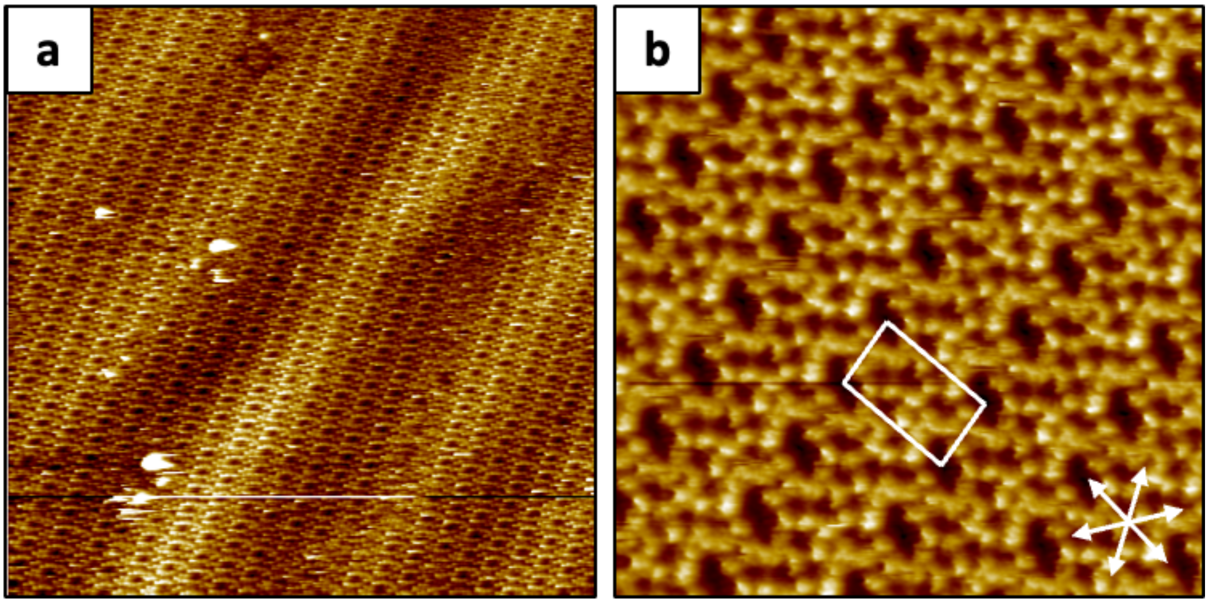

Figure S1. Additional STM images of Y6 on HOPG measured at the solid-liquid interface (HOPG/1-PO). **a)** Large area STM image of a monodomain (scanned area: 100 nm × 100 nm; *I* = 101 pA V = 0.95 V). **b)** HR-STM image of a zoomed area (scanned area: 20 nm × 20 nm; *I* = 101 pA V = 0.95 V).

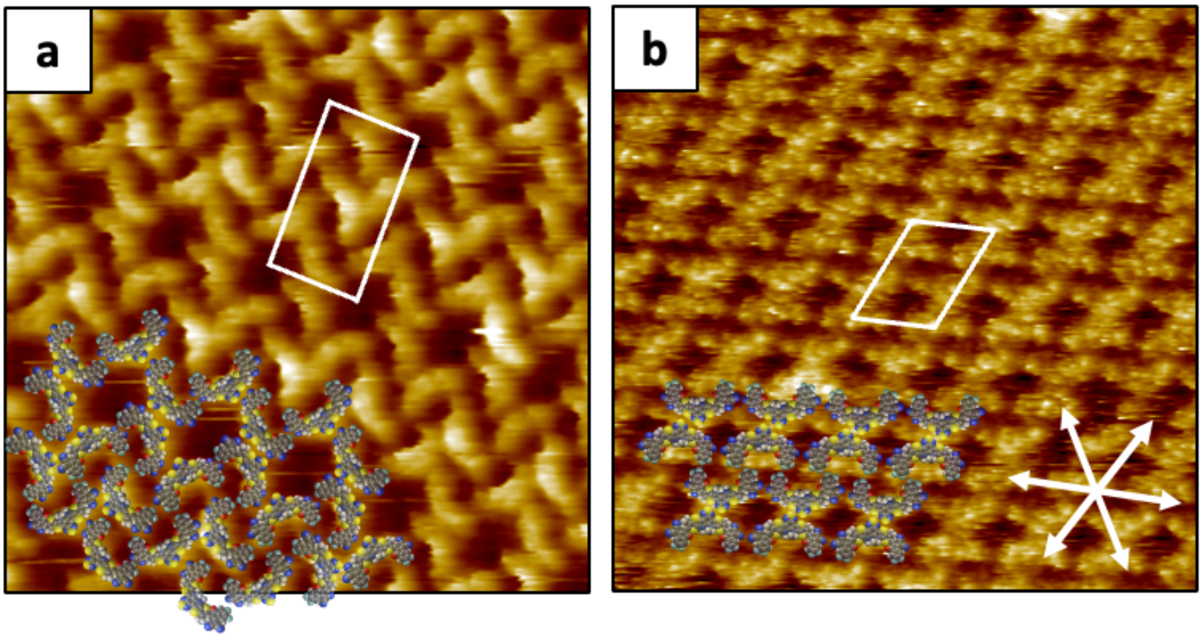

Figure S2. STM images of $P1_{Y6}$ and $P2_{Y6}$ showed in **Figure 2** of the main text. Upon geometry optimization by DFT calculations, individual Y6 molecules were scaled and manually superimposed to the STM images to reveal the spatial arrangement of the molecules within the supramolecular structures. **a)** HR-STM image of the $P1_{Y6}$ consisting of five Y6 molecules per unit cell organized in a nanoporous network (scanned area: 20 nm × 20 nm; *I* = 43 pA V = 1.35 V). **b)** HR-STM image of $P1_{Y6}$ consisting of two Y6 molecules in a dimer-like supramolecular structure (scanned area: 20 nm × 20 nm; *I* = 43 pA V = 1.35 V).

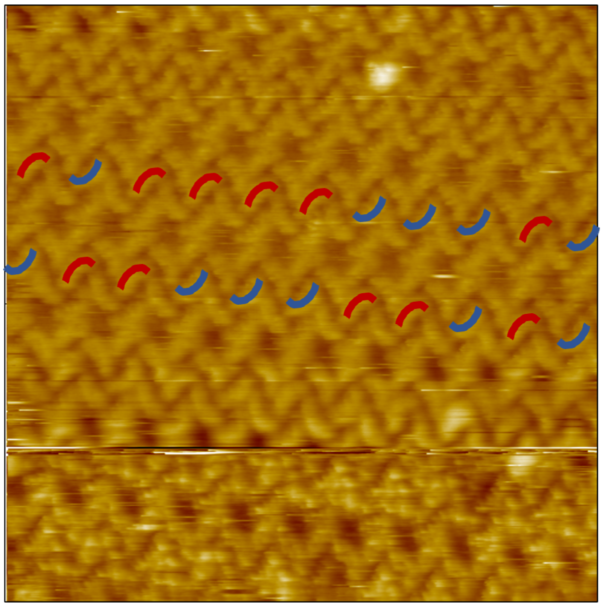

Figure S3. HR-STM image of P1_{Y6}. In the STM image the individual Y6 molecules that can freely rotate within the supramolecular network are highlighted in blue and red (scanned area: 30 nm × 30 nm; *I* = 49 pA V = 1.35 V).

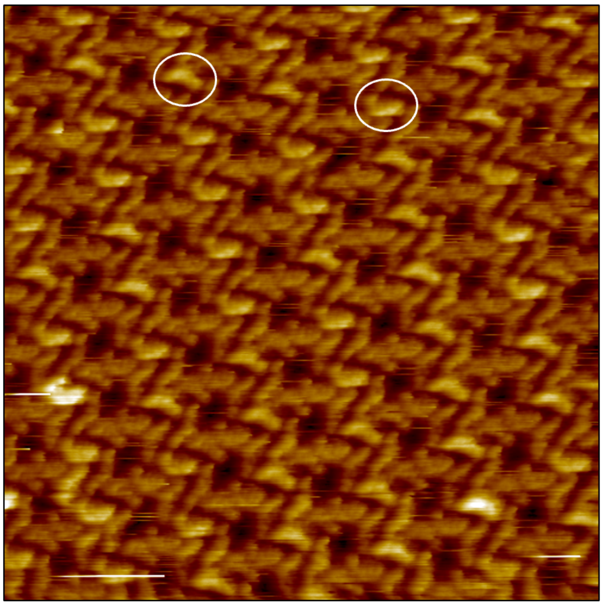

Figure S4. HR-STM image of the P1_{Y6} after pulsing with high voltage the STM tip. The central Y6 molecules of each unit cell appeared brighter in the STM contrast probably due to the bending out-of-plane of the long alkyl chains (in the image some Y6 molecules are highlighted within the white circles). Scanned area: 30 nm × 30 nm; *I* = 40 pA V = 1.35 V.

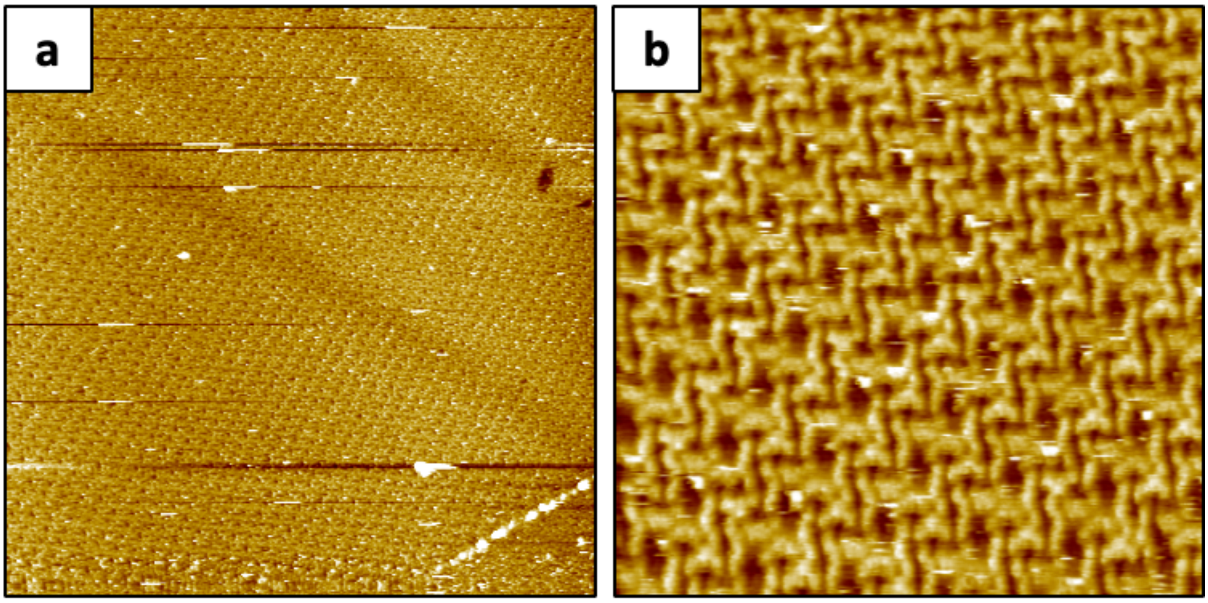

Figure S5. Two representative STM images of a 2D SAMN composed of Y6 molecules measured at negative tip bias at the solid-liquid interface (HOPG/1-PO). **a)** Scanned area: 150 nm × 150 nm; *I* = –49 pA V = –1.35 V, and **b)** scanned area: 30 nm × 30 nm; *I* = –49 pA V = –1.35 V.

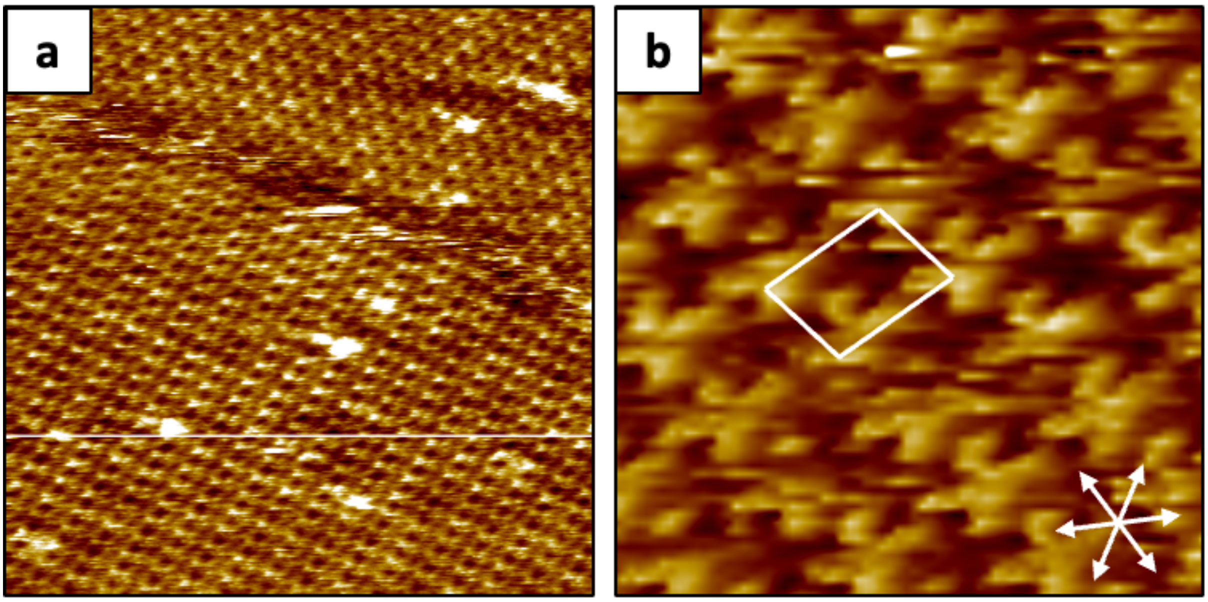

Figure S6. STM images of a 2D SAMN composed of Y6 molecules measured by STM at the **a)** HOPG/octanoic acid interface (scanned area: 30 nm × 30 nm; *I* = 64 pA V = 1.35 V), and **b)** HOPG/1,2,4-trichlorobenzene interface (scanned area: 12 nm × 12 nm; *I* = 189 pA V = 1.2 V).

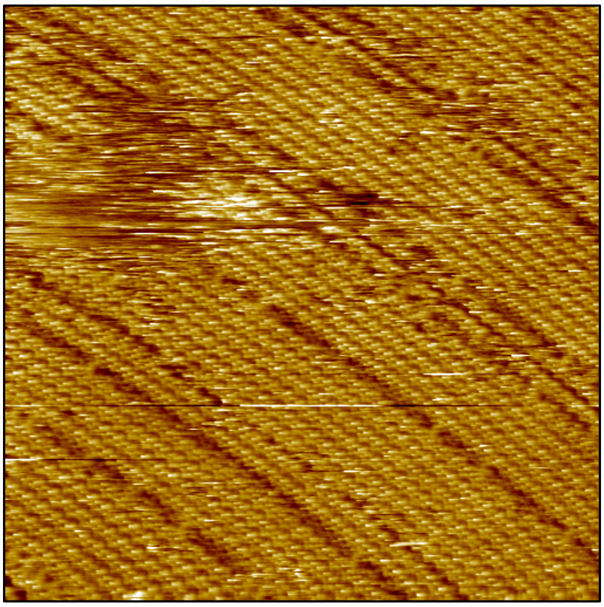

Figure S7. Large area STM image of Y6:HBC interfacial cocrystals (scanned area: 100 nm × 100 nm; *I* = 101 pA V = 0.9 V). The large scale STM image revealed the overall of Y6 and HBC molecules in quasi 1D rows on the HOPG surface.

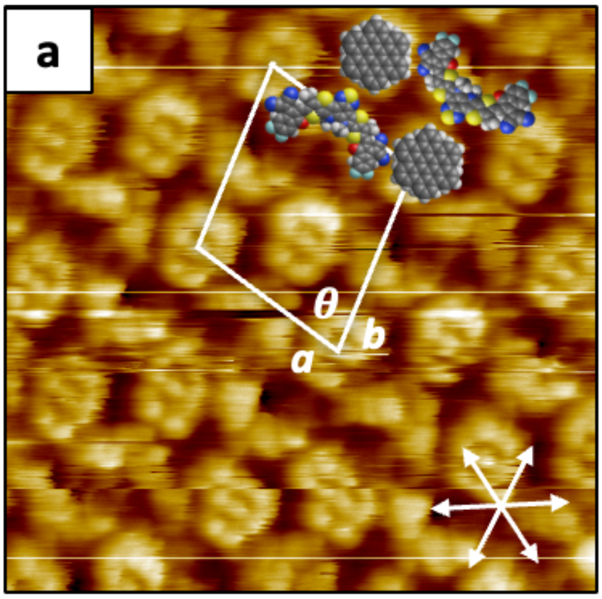

Figure S8. HR-STM image of a defect-free Y6:HBC interfacial cocrystal as shown in **Figure 4a** of the main text (scanned area: 10 nm × 10 nm; *I* = 79 pA V = 0.9 V). Y6 and HBC structures were simulated by DFT calculations, scaled and manually superimposed on the STM image to reveal the spatial arrangement of the molecules.

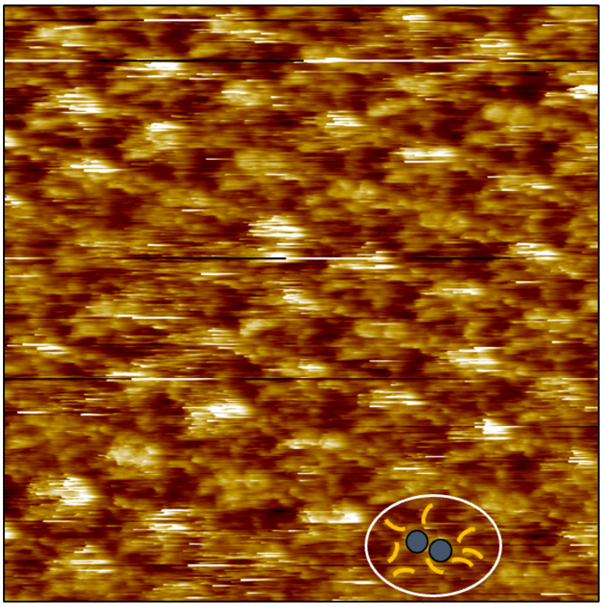

Figure S9. HR-STM image of the second polymorph P2$_{Y6:HBC}$ interfacial cocrystal measured at the solid-liquid interface (HOPG/1-PO; scanned area: 30 nm × 30 nm; *I* = 49 pA V = 1.35 V). Here, two HBC molecules (highlighted with grey circles) form a closely interacting supramolecular dimer that is surrounded by Y6 molecules (highlighted with yellow lines).

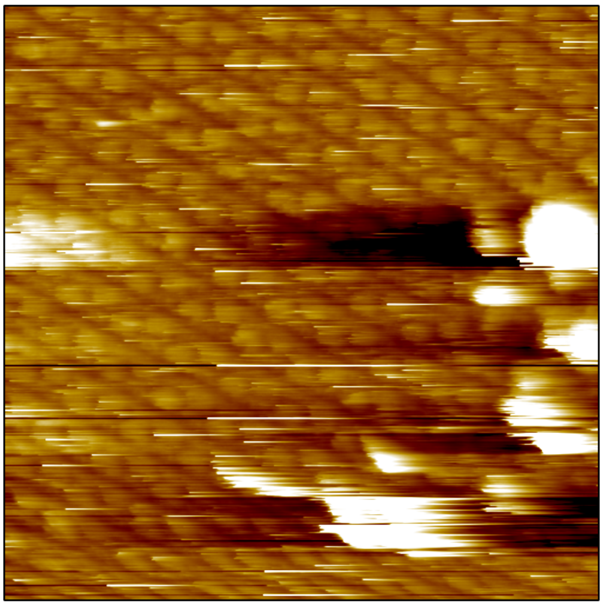

Figure S10. STM image of the first polymorph P1$_{Y6:HBC}$ interfacial cocrystal measured on HOPG using octanoic acid as STM solvent. Scanned area: 30 nm × 30 nm; *I* = 52 pA V = 1.35 V.

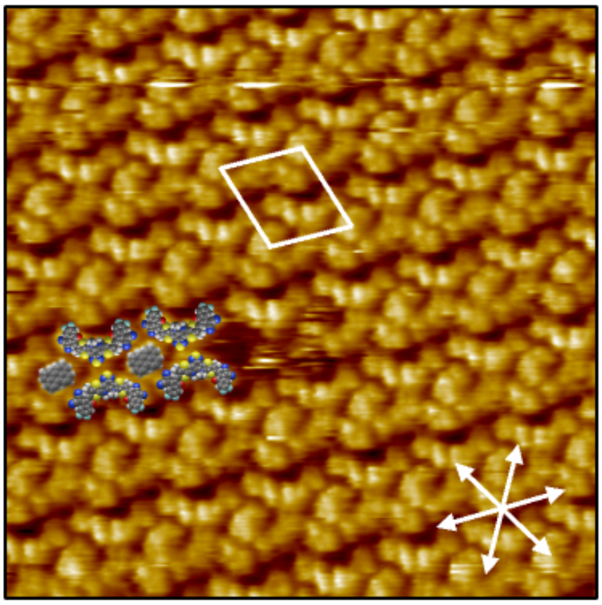

Figure S11. HR-STM image of a Y6:perylene interfacial cocrystal as shown in **Figure 5a** of the main text (scanned area: 20 nm × 20 nm; *I* = 49 pA V = 1.35 V). Y6 and perylene structures were simulated by DFT calculations, scaled and superimposed on the STM image to reveal the spatial arrangement of the molecules.

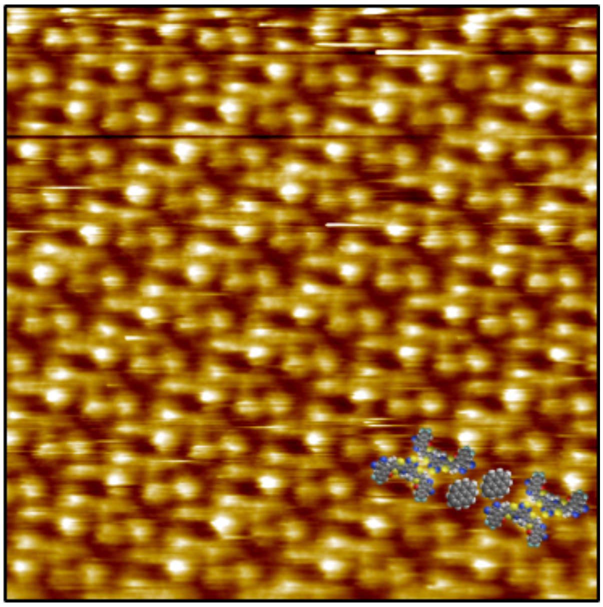

Figure S12. HR-STM image of a Y6:pyrene interfacial cocrystal (scanned area: 20 nm × 20 nm; *I* = 58 pA V = 1.05 V). Y6 and pyrene structures were simulated by DFT calculations, scaled and superimposed on the STM image to reveal the spatial arrangement of the molecules.

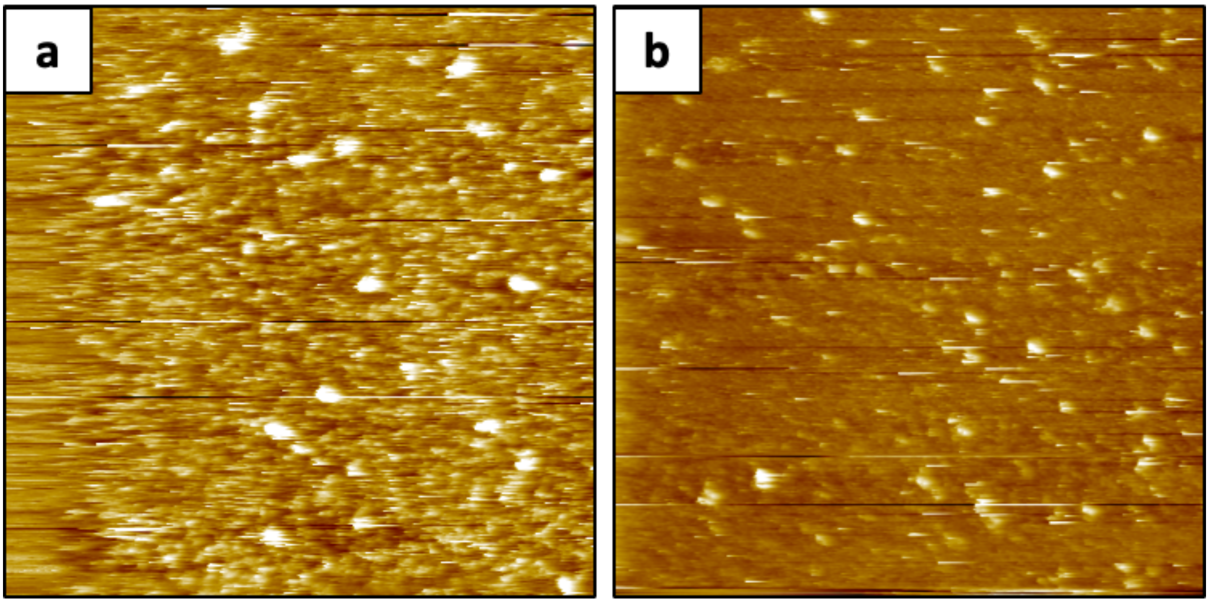

Figure S13. STM images obtained upon deposition of a premixed solution of Y6 with a saturated solution of **a)** coronene (scanned area: 60 nm × 60 nm; *I* = 49 pA V = 1.25 V), and **b)** benzo[*ghi*]perylene (scanned area: 100 nm × 100 nm; *I* = 58 pA V = 1.15 V). Only amorphous material could be observed for both cases.

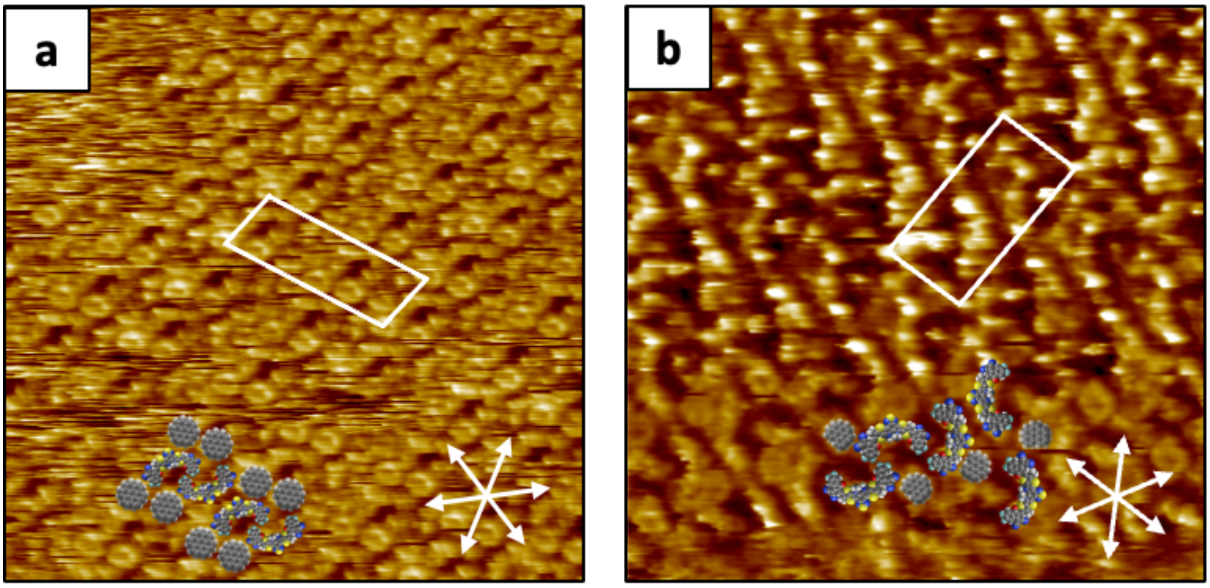

Figure S14. HR-STM image of Y6:PAHs interfacial cocrystals. **a)** Y6:coronene (scanned area: 20 nm × 20 nm; *I* = 49 pA V = 1.35 V), and **b)** Y6:benzo[*ghi*]perylene cocrystals (scanned area: 20 nm × 20 nm; *I* = 49 pA V = 1.35 V). Coronene and benzo[*ghi*]perylene structures were simulated by DFT calculations, scaled and superimposed on the STM image to reveal the spatial arrangement of the molecules.

DFT Calculations

Investigated molecules

The molecules investigated in this study are the truncated Y6, coronene, benzo[*ghi*]perylene, hexabenzocoronene, perylene, and pyrene. Their HOMO and LUMO orbital energies are listed below in **Table S1**. The orbitals depicted were rendered in Avogadro using an isodensity value of 0.02.

Table S1: DFT calculated HOMO–LUMO values of the investigated molecular systems.

Molecule	HOMO [eV]	LUMO [eV]
Y6 truncated	-7.822	-2.201
Coronene	-7.826	0.070
Benzo[*ghi*]perylene	-7.607	-0.171
Hexabenzocoronene	-7.589	-0.218
Perylene	-7.368	-0.408
Pyrene	-7.792	0.089

Adiabatic IP and EA

To gain better insight into the electronic properties of the molecular systems, adiabatic ionization potential and adiabatic electron affinity was calculated using the same methodology as described above. The results were obtained using zero-point energies.

Table S2: DFT calculated adiabatic ionization potential (IP) and electron affinity (EA) of the investigated molecular systems. The energy gap (IP – EA) is reported as well.

Molecule	IP [eV]	EA [eV]	Gap [eV]
Benzo[*ghi*]perylene	7.256	0.802	6.454
Coronene	7.416	0.635	6.781
Hexabenzocoronene	7.126	0.986	6.140
Perylene	7.003	1.049	5.955
Pyrene	7.454	0.546	6.908
Truncated Y6	7.021	3.037	3.984

Table S3: Energies used for band gap calculation

Molecule	SCF [Eh]	ZPE total [Eh]	ZPE correction [Eh]
Benzo[*ghi*]perylene	-132.560918	-132.2871006	0.27381744
Benzo[*ghi*]perylene$^{\cdot -}$	-132.5851233	-132.3165665	0.26855677
Benzo[*ghi*]perylene$^{\cdot +}$	-132.2939156	-132.020445	0.27347062
Coronene	-143.9731811	-143.6859698	0.28721123
Coronene$^{\cdot -}$	-143.9881166	-143.709315	0.27880163
Coronene$^{\cdot +}$	-143.6976069	-143.4134257	0.28418126
Hexabenzocoronene	-250.1519884	-249.6806011	0.47138728
Hexabenzocoronene$^{\cdot -}$	-250.1810719	-249.7168178	0.4642541
Hexabenzocoronene$^{\cdot +}$	-249.8881006	-249.418728	0.46937265
Perylene	-121.1422476	-120.882034	0.26021364
Perylene$^{\cdot -}$	-121.1760179	-120.9205771	0.2554408
Perylene$^{\cdot +}$	-120.885003	-120.6246603	0.26034264
Pyrene	-97.16252395	-96.95015919	0.21236476
Pyrene$^{\cdot -}$	-97.177101	-96.97023702	0.20686398
Pyrene$^{\cdot +}$	-96.88824642	-96.67622624	0.21202018
Truncated Y6	-544.1280819	-543.5609843	0.56709754
Truncated Y6$^{\cdot -}$	-544.2360356	-543.6726071	0.56342844
Truncated Y6$^{\cdot +}$	-543.8696464	-543.3029687	0.56667768

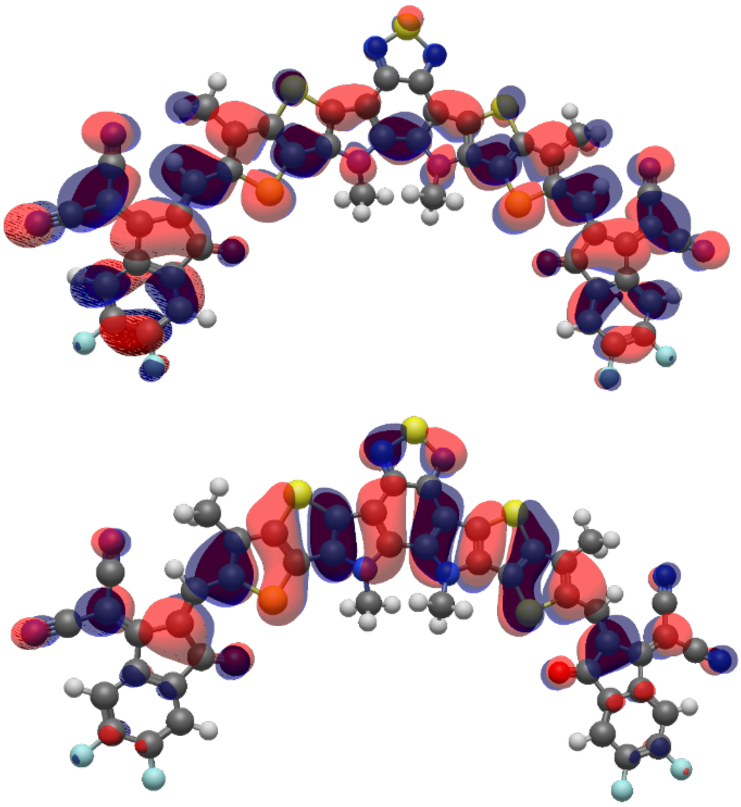

Figure S15: Truncated Y6 with HOMO (bottom) and LUMO (top) orbitals rendered.

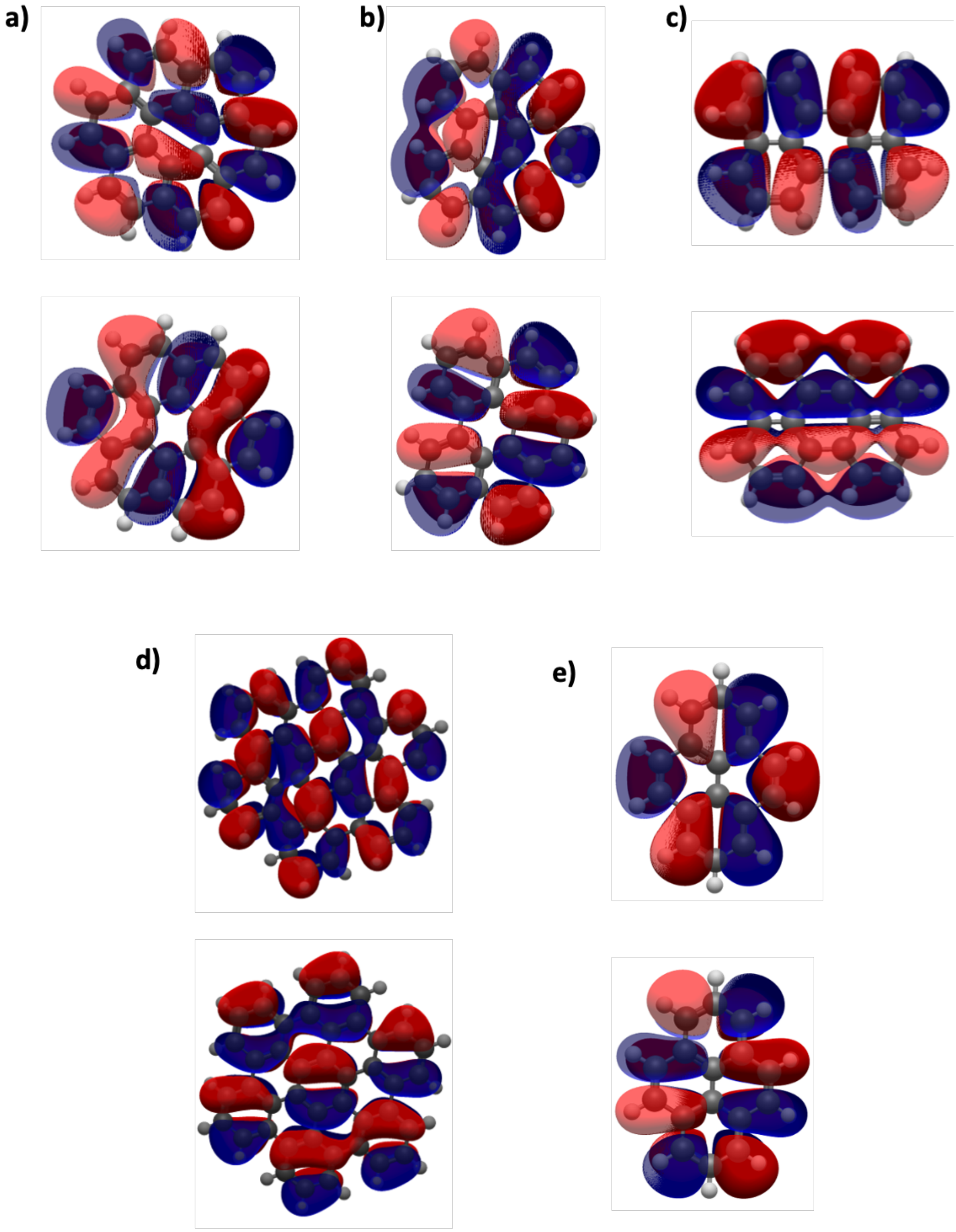

Figure S16: DFT simulated **a)** coronene, **b)** benzo[*ghi*]perylene, **c)** perylene, **d)** HBC, and **e)** pyrene structures with HOMO (bottom) and LUMO (top) orbitals rendered.

Table S4: Coordinates of the optimized structures

Y6 geometries											
Neutral				Radical anion				Radical cation			
6	-3.579147	10.414822	4.336527	6	-3.601539	10.441103	4.351998	6	-3.542467	10.423977	4.307971
6	-3.60555	10.667062	2.933315	6	-3.621665	10.688646	2.949758	6	-3.553191	10.688343	2.901173
6	-2.775137	9.949421	2.07085	6	-2.79222	9.948734	2.088745	6	-2.722416	9.93362	2.012798
6	-1.991081	8.864622	2.57485	6	-2.024234	8.868892	2.59219	6	-1.996583	8.851213	2.497383
6	-1.891807	8.659389	3.951968	6	-1.923409	8.661476	3.977437	6	-1.913395	8.62471	3.908335
6	-2.670986	9.43972	4.855992	6	-2.703604	9.452863	4.875416	6	-2.67729	9.42187	4.819424
6	-4.252972	11.639113	2.147755	6	-4.249705	11.660008	2.161348	6	-4.197668	11.634212	2.142276
6	-3.779008	11.504481	0.861187	6	-3.781059	11.51374	0.866685	6	-3.735779	11.49707	0.821227
7	-2.866782	10.478872	0.801797	7	-2.879506	10.47165	0.817865	7	-2.836824	10.478454	0.742722
16	-5.385862	12.929659	2.337845	16	-5.374745	12.955751	2.34874	16	-5.322723	12.931046	2.348505
6	-5.20141	13.300731	0.640281	6	-5.198173	13.317755	0.645305	6	-5.150555	13.29321	0.645227
6	-4.314329	12.464579	-0.021229	6	-4.310434	12.460081	-0.013686	6	-4.274993	12.456498	-0.038075
6	-5.779644	14.266659	-0.183681	6	-5.764778	14.273308	-0.174079	6	-5.739796	14.267335	-0.161568
6	-5.312561	14.15622	-1.494966	6	-5.301711	14.157	-1.501815	6	-5.290935	14.153681	-1.477103
16	-4.164584	12.840976	-1.677763	16	-4.159512	12.825133	-1.673984	16	-4.157742	12.848428	-1.701893
7	-1.270239	7.89318	1.914502	7	-1.277875	7.898516	1.942887	7	-1.261312	7.86644	1.855018
6	-0.692091	7.100812	2.876597	6	-0.695362	7.119915	2.908869	6	-0.702726	7.086986	2.820655
6	-1.047183	7.547741	4.130458	6	-1.064617	7.574701	4.165557	6	-1.083792	7.54624	4.093944
6	0.115748	5.946289	2.917007	6	0.102367	5.96755	2.94206	6	0.094095	5.941904	2.879601
6	0.357936	5.560564	4.227019	6	0.346051	5.568826	4.249712	6	0.316879	5.56428	4.199176
16	-0.398397	6.593415	5.416304	16	-0.416031	6.600639	5.446168	16	-0.449344	6.592754	5.38951
16	0.876694	4.928981	1.779269	16	0.884675	4.94227	1.79995	16	0.879539	4.908961	1.760672
6	1.537541	3.950504	3.078177	6	1.543721	3.950556	3.103996	6	1.507217	3.955	3.077641
6	1.166352	4.428865	4.336414	6	1.152868	4.443466	4.36855	6	1.121817	4.432081	4.33019
6	-5.777151	15.048945	-2.487395	6	-5.757667	15.035591	-2.483604	6	-5.77994	15.067265	-2.469167
6	-5.533602	15.213636	-3.816262	6	-5.51716	15.196989	-3.829605	6	-5.553256	15.227812	-3.788004
6	-6.133703	16.22302	-4.685722	6	-6.113959	16.198758	-4.68741	6	-6.158494	16.244655	-4.667397
6	-5.565094	16.022836	-6.039488	6	-5.556857	15.997442	-6.050739	6	-5.593907	16.034397	-6.01566
6	-4.677768	14.959359	-6.002298	6	-4.673822	14.931085	-6.016152	6	-4.709955	14.965117	-5.982835
6	-4.616252	14.410145	-4.635812	6	-4.610606	14.388385	-4.642287	6	-4.640621	14.416175	-4.62577
6	-5.777036	16.691557	-7.236492	6	-5.773459	16.66305	-7.246935	6	-5.809156	16.704526	-7.212102
6	-5.083815	16.25661	-8.341675	6	-5.087969	16.222109	-8.356387	6	-5.124107	16.266526	-8.320334
6	-4.197854	15.187677	-8.285358	6	-4.207319	15.152037	-8.302664	6	-4.24068	15.190747	-8.269122
6	-3.981837	14.520248	-7.105834	6	-3.986762	14.486432	-7.121065	6	-4.021363	14.522561	-7.092153
6	2.353717	2.801379	2.972602	6	2.34712	2.815116	2.991298	6	2.339069	2.789277	2.994121
6	2.879636	2.093039	1.936281	6	2.890937	2.094508	1.911202	6	2.872786	2.090189	1.972947
6	2.707263	2.389517	0.507589	6	2.725055	2.418401	0.499904	6	2.715864	2.387678	0.530648
6	3.457664	1.358858	-0.232288	6	3.472016	1.394071	-0.269695	6	3.471612	1.36491	-0.197913
6	4.053877	0.483043	0.660588	6	4.052526	0.50687	0.624582	6	4.062654	0.485778	0.698532
6	3.718854	0.901716	2.042073	6	3.694717	0.934561	2.006841	6	3.721321	0.8892	2.077379
6	3.579348	1.240429	-1.598311	6	3.602497	1.290204	-1.629343	6	3.606349	1.250339	-1.565139
6	4.33361	0.194886	-2.068763	6	4.356031	0.239153	-2.110146	6	4.366294	0.208661	-2.031212
6	4.939066	-0.693418	-1.188392	6	4.941511	-0.653992	-1.237672	6	4.967012	-0.683245	-1.145757
6	4.814593	-0.571045	0.175685	6	4.805696	-0.543335	0.134305	6	4.830246	-0.565706	0.216856

6	-7.042432	17.181991	-4.37109	6	-7.020508	17.170391	-4.375556	6	-7.06082	17.197334	-4.339773
6	4.140633	0.257789	3.160954	6	4.123419	0.245915	3.142344	6	4.127056	0.252134	3.199251
7	-2.706128	9.339464	6.165933	7	-2.744565	9.357923	6.184887	7	-2.720203	9.308248	6.125342
16	-3.811366	10.408306	6.698942	16	-3.839431	10.43929	6.715313	16	-3.793224	10.405272	6.664726
7	-4.275549	11.027651	5.267506	7	-4.29471	11.063853	5.278222	7	-4.225933	11.050495	5.235656
6	-1.884418	10.391635	-0.259968	6	-1.892343	10.365255	-0.236459	6	-1.901387	10.362898	-0.362687
6	-1.462655	7.42639	0.556223	6	-1.467922	7.40358	0.595318	6	-1.392209	7.41214	0.481434
8	2.081037	3.293035	0.007661	8	2.109782	3.333713	-0.007221	8	2.087603	3.296393	0.046016
8	-3.932681	13.477812	-4.286623	8	-3.925752	13.452873	-4.298405	8	-3.967544	13.482983	-4.263195
6	-7.586773	17.366803	-3.067421	6	-7.560944	17.369044	-3.074148	6	-7.589553	17.37112	-3.026529
7	-8.041397	17.537736	-2.025467	7	-8.01708	17.557149	-2.034832	7	-8.021766	17.52044	-1.972727
6	-7.554074	18.114085	-5.320635	6	-7.529471	18.099064	-5.327129	6	-7.582051	18.133421	-5.282299
7	-7.987102	18.880382	-6.059341	7	-7.962674	18.865775	-6.066877	7	-8.018381	18.899047	-6.018229
6	3.812523	0.665424	4.486168	6	3.803472	0.611865	4.472359	6	3.777303	0.667813	4.518025
7	3.562603	0.972542	5.56542	7	3.571671	0.867171	5.573998	7	3.501617	0.993866	5.584459
6	4.963666	-0.905711	3.128505	6	4.93611	-0.911618	3.084407	6	4.953088	-0.911253	3.178681
7	5.626492	-1.844304	3.139343	7	5.59824	-1.857113	3.074094	7	5.616942	-1.847873	3.192851
1	-6.407215	16.291093	0.152701	1	-6.373622	16.304603	0.140817	1	-6.351517	16.294501	0.190931
6	-6.765856	15.274512	0.301403	6	-6.741814	15.293191	0.301423	6	-6.714469	15.27951	0.339498
6	1.563629	3.828372	5.642287	6	1.553781	3.828316	5.665045	6	1.502309	3.833987	5.642847
1	-6.482715	15.74342	-2.062208	1	-6.457963	15.739036	-2.063977	1	-6.475681	15.759318	-2.025975
1	-6.447004	17.526328	-7.354689	1	-6.44115	17.499959	-7.362852	1	-6.475734	17.54267	-7.326967
9	-5.256556	16.868166	-9.506315	9	-5.267389	16.834105	-9.523829	9	-5.298145	16.875285	-9.479643
9	-3.565771	14.825481	-9.393774	9	-3.581187	14.782514	-9.415543	9	-3.619354	14.828334	-9.377704
1	-3.29544	13.68806	-7.050494	1	-3.303442	13.651478	-7.066329	1	-3.337924	13.687621	-7.043812
1	2.596497	2.426263	3.953024	1	2.59253	2.418773	3.96119	1	2.564282	2.42227	3.980998
1	3.104233	1.936947	-2.273496	1	3.136931	1.998775	-2.299082	1	3.13721	1.946281	-2.244953
9	4.499115	0.012242	-3.372049	9	4.527168	0.074382	-3.428055	9	4.544143	0.029715	-3.328389
9	5.657552	-1.687364	-1.694582	9	5.664038	-1.659671	-1.743333	9	5.688469	-1.66923	-1.648112
1	5.311867	-1.296432	0.79713	1	5.291386	-1.277902	0.754632	1	5.323512	-1.292309	0.840354
1	-2.221653	9.75724	-1.076464	1	-2.228075	9.722426	-1.047053	1	-2.263573	9.684944	-1.13063
1	-0.949967	10.011864	0.138028	1	-0.964596	9.98066	0.172759	1	-0.939739	10.028105	0.009263
1	-0.646989	7.726217	-0.097716	1	-0.661605	7.705907	-0.069621	1	-0.57659	7.767584	-0.142272
1	-2.401698	7.806272	0.169117	1	-2.417325	7.755495	0.205224	1	-2.342932	7.742859	0.07908
1	-7.717658	15.186415	-0.218718	1	-7.692661	15.208844	-0.221197	1	-7.674103	15.197711	-0.166856
1	-6.955139	15.142304	1.363172	1	-6.932794	15.172148	1.364297	1	-6.892515	15.147473	1.403276
1	2.644944	3.806414	5.761948	1	2.636717	3.804294	5.781609	1	2.581637	3.818308	5.779562
1	1.150921	4.402753	6.467215	1	1.139823	4.388123	6.501183	1	1.076273	4.405428	6.463088
1	-1.69665	11.389837	-0.647318	1	-1.694432	11.357572	-0.634388	1	-1.75883	11.347116	-0.800005
1	-1.523764	6.341074	0.563566	1	-1.499575	6.317171	0.623428	1	-1.393558	6.325743	0.474537
1	1.205271	2.805313	5.738814	1	1.207282	2.798591	5.747133	1	1.1459	2.810057	5.736051

Coronene

Neutral				Radical anion				Radical cation			
6	1.776993	1.282892	-5.846747	6	1.776967	1.273796	-5.866336	6	1.777028	1.290485	-5.85858
6	1.777001	2.222925	-4.786658	6	1.777002	2.231995	-4.801246	6	1.776997	2.226797	-4.797509
6	1.777009	1.757468	-3.468909	6	1.777018	1.752658	-3.466391	6	1.776963	1.754053	-3.459129
6	1.777006	0.363237	-3.209306	6	1.777017	0.363808	-3.208577	6	1.776957	0.372345	-3.202291

6	1.776997	-0.545258	-4.271284	6	1.776984	-0.560636	-4.289351	6	1.776971	-0.542367	-4.270003
6	1.776991	-0.049695	-5.598648	6	1.776965	-0.058662	-5.607904	6	1.777007	-0.040831	-5.607893
6	1.777007	2.679355	-2.391319	6	1.777005	2.676153	-2.386921	6	1.776946	2.667881	-2.391634
6	1.777006	-0.109065	-1.872144	6	1.777036	-0.105863	-1.876544	6	1.77697	-0.097594	-1.871824
6	1.777006	0.81282	-0.794555	6	1.777027	0.817626	-0.797076	6	1.77698	0.81623	-0.804321
6	1.777005	2.207051	-1.054157	6	1.777009	2.206476	-1.054894	6	1.776959	2.197936	-1.061166
6	1.776998	0.347359	0.523194	6	1.777019	0.338289	0.53778	6	1.77701	0.343491	0.534058
6	1.776988	-1.049963	0.757787	6	1.777033	-1.03546	0.765206	6	1.777013	-1.052452	0.771832
6	1.77699	-1.931146	-0.272184	6	1.777043	-1.936239	-0.287872	6	1.77699	-1.930628	-0.258778
6	1.776999	-1.483039	-1.61631	6	1.777029	-1.498928	-1.609551	6	1.776978	-1.480316	-1.615381
6	1.776991	-2.38479	-2.709126	6	1.776982	-2.402929	-2.721352	6	1.776972	-2.367279	-2.687634
6	1.776991	-1.933356	-3.987221	6	1.776957	-1.941573	-3.99738	6	1.776969	-1.906296	-3.992513
1	1.776989	-2.633124	-4.812225	1	1.776906	-2.642559	-4.824752	1	1.776971	-2.614244	-4.809813
1	1.776985	-3.447448	-2.506591	1	1.776951	-3.467135	-2.521635	1	1.776972	-3.431464	-2.496342
1	1.776981	1.647547	-6.865247	1	1.776967	1.635827	-6.88681	1	1.77707	1.654837	-6.875932
1	1.776986	-0.756413	-6.4177	1	1.776972	-0.767536	-6.428525	1	1.777039	-0.748338	-6.425686
1	1.776974	-1.405815	1.779386	1	1.777026	-1.39596	1.786644	1	1.77703	-1.407515	1.792453
1	1.776977	-2.995508	-0.078785	1	1.777029	-3.00118	-0.089872	1	1.776991	-2.994835	-0.067088
6	1.776993	3.620247	-5.021251	6	1.777024	3.605747	-5.028666	6	1.777	3.622738	-5.03531
6	1.776994	4.501432	-3.991281	6	1.777027	4.506532	-3.975592	6	1.77699	4.500907	-4.004696
6	1.776998	4.053331	-2.647154	6	1.777	4.069222	-2.653912	6	1.776968	4.050598	-2.64809
1	1.776983	3.976095	-6.04285	1	1.777032	3.966239	-6.050099	1	1.77701	3.977792	-6.055935
1	1.776989	5.565791	-4.18469	1	1.77705	5.571477	-4.173594	1	1.776993	5.565113	-4.196369
6	1.776988	4.95508	-1.554338	6	1.776975	4.973222	-1.542114	6	1.776975	4.937561	-1.575843
6	1.776989	4.503644	-0.276243	6	1.776958	4.511859	-0.266089	6	1.77698	4.476582	-0.270962
6	1.776997	3.115547	0.007819	6	1.776975	3.130921	0.025877	6	1.776978	3.112654	0.006542
1	1.776975	6.017737	-1.756872	1	1.776965	6.037427	-1.741824	1	1.776988	6.001743	-1.767145
1	1.776975	5.203414	0.548759	1	1.776953	5.212841	0.561291	1	1.776981	5.184543	0.546328
6	1.776997	1.287392	1.583281	6	1.776982	1.296488	1.602868	6	1.777022	1.279806	1.595129
1	1.776996	0.922739	2.601783	1	1.77696	0.934451	2.623338	1	1.777035	0.915473	2.612495
6	1.776996	2.61998	1.335184	6	1.776963	2.628948	1.344435	6	1.777004	2.611126	1.344434
1	1.776994	3.326698	2.154235	1	1.77693	3.337816	2.165072	1	1.777027	3.318628	2.16223

Benzo[*ghi*]perylene

Neutral				Radical anion				Radical cation			
6	1.777007	1.292167	-5.810804	6	1.776976	1.282562	-5.835791	6	1.776945	1.281741	-5.831688
6	1.777001	2.221906	-4.754769	6	1.776992	2.232461	-4.775158	6	1.776966	2.223431	-4.761765
6	1.776988	1.761676	-3.436088	6	1.776985	1.760337	-3.43969	6	1.776962	1.757068	-3.429476
6	1.776978	0.37113	-3.177162	6	1.776958	0.371072	-3.181	6	1.776964	0.377806	-3.172659
6	1.776979	-0.532835	-4.241882	6	1.776957	-0.550031	-4.257111	6	1.776962	-0.536798	-4.247875
6	1.776994	-0.045433	-5.561762	6	1.776955	-0.045456	-5.588534	6	1.776938	-0.043238	-5.585003
6	1.776983	2.690105	-2.352267	6	1.777006	2.683422	-2.356412	6	1.776973	2.675273	-2.355138
6	1.776978	-0.105012	-1.831818	6	1.776972	-0.10025	-1.838095	6	1.776974	-0.092186	-1.839853
6	1.776994	0.797421	-0.746897	6	1.776986	0.812869	-0.754799	6	1.776931	0.811192	-0.758109
6	1.776978	2.238614	-1.015291	6	1.776986	2.22139	-1.017104	6	1.776944	2.221769	-1.020785
6	1.777025	0.282374	0.536742	6	1.776988	0.288087	0.557534	6	1.776917	0.285085	0.547945
6	1.777029	-1.086314	0.767872	6	1.777005	-1.071789	0.777853	6	1.776975	-1.070107	0.778298

6	1.776997	-1.967818	-0.28372	6	1.777009	-1.971975	-0.271297	6	1.777041	-1.953921	-0.285942
6	1.776977	-1.492898	-1.595071	6	1.776991	-1.502482	-1.596072	6	1.77703	-1.481241	-1.600817
6	1.776968	-2.392706	-2.706207	6	1.777002	-2.388531	-2.698382	6	1.777058	-2.373706	-2.696176
6	1.77697	-1.934719	-3.970091	6	1.776987	-1.922851	-3.984419	6	1.77701	-1.912993	-3.981607
1	1.776966	-2.622856	-4.804763	1	1.777007	-2.618929	-4.815085	1	1.777001	-2.609888	-4.807847
1	1.77696	-3.45563	-2.505767	1	1.777043	-3.453626	-2.502561	1	1.77712	-3.437421	-2.502188
1	1.777022	1.657776	-6.828898	1	1.776966	1.649465	-6.855441	1	1.776943	1.651815	-6.847232
1	1.776991	-0.752911	-6.380075	1	1.776954	-0.754697	-6.407837	1	1.776934	-0.753969	-6.399328
1	1.777058	-1.454361	1.784343	1	1.777017	-1.440996	1.796886	1	1.776973	-1.443353	1.791701
1	1.776997	-3.034895	-0.107128	1	1.777036	-3.03861	-0.087487	1	1.777105	-3.020368	-0.106627
6	1.777013	3.627677	-5.005767	6	1.777012	3.61145	-5.014846	6	1.777001	3.603248	-5.008683
6	1.777004	4.509881	-3.991418	6	1.777026	4.508811	-3.982622	6	1.777031	4.495761	-3.975229
6	1.77699	4.070215	-2.630894	6	1.777019	4.078814	-2.635276	6	1.777014	4.057293	-2.632055
1	1.777031	3.96918	-6.032206	1	1.777021	3.961781	-6.040412	1	1.777023	3.955919	-6.030416
1	1.777014	5.573734	-4.186907	1	1.777057	5.573015	-4.183252	1	1.777064	5.558032	-4.177031
6	1.776982	4.985186	-1.578276	6	1.777009	4.993536	-1.568178	6	1.777038	4.971433	-1.575322
6	1.776967	4.541206	-0.27989	6	1.776971	4.531238	-0.265331	6	1.777016	4.529846	-0.264296
6	1.776962	3.181042	-0.003027	6	1.776965	3.183178	0.018427	6	1.776963	3.182539	0.00843
1	1.776986	6.044339	-1.797431	1	1.777008	6.054871	-1.780459	1	1.777076	6.030984	-1.79169
1	1.776955	5.250363	0.536049	1	1.776954	5.242392	0.552581	1	1.77703	5.242767	0.546905
1	1.777054	0.942615	1.389431	1	1.776986	0.951455	1.407493	1	1.776879	0.946214	1.399237
1	1.776949	2.87172	1.030018	1	1.776942	2.869954	1.050055	1	1.776949	2.871913	1.040497

Hexabenzocoronene

Neutral				Radical anion				Radical cation			
6	-0.403387	-0.142179	0.056038	6	-0.405403	-0.142615	0.05821	6	-0.403387	-0.142179	0.056038
6	-1.735474	-0.635102	-0.042949	6	-1.736724	-0.641564	-0.037496	6	-1.735474	-0.635102	-0.042949
6	-2.833286	0.271962	0.001679	6	-2.831766	0.270814	0.003656	6	-2.833286	0.271962	0.001679
6	-2.60179	1.641893	0.084692	6	-2.599938	1.642017	0.08755	6	-2.60179	1.641893	0.084692
6	-1.267965	2.137898	0.126764	6	-1.26713	2.143595	0.133742	6	-1.267965	2.137898	0.126764
6	-0.172073	1.227688	0.140427	6	-0.173757	1.22856	0.142833	6	-0.172073	1.227688	0.140427
6	-3.702161	2.554234	0.151382	6	-3.706167	2.555276	0.148674	6	-3.702161	2.554234	0.151382
6	-3.471826	3.941489	0.173799	6	-3.473561	3.950798	0.188032	6	-3.471826	3.941489	0.173799
6	-2.113715	4.443038	0.055401	6	-2.108857	4.451679	0.079706	6	-2.113715	4.443038	0.055401
6	-1.02956	3.535787	0.119346	6	-1.030316	3.533345	0.127362	6	-1.02956	3.535787	0.119346
6	0.295508	4.032682	0.114509	6	0.292588	4.042574	0.136162	6	0.295508	4.032682	0.114509
6	1.404086	3.110634	0.293594	6	1.408376	3.118695	0.304203	6	1.404086	3.110634	0.293594
6	1.163537	1.724978	0.265718	6	1.167671	1.724489	0.26165	6	1.163537	1.724978	0.265718
6	2.240562	0.830906	0.387011	6	2.25344	0.831896	0.356913	6	2.240562	0.830906	0.387011
6	2.002348	-0.587153	0.289954	6	2.014905	-0.588201	0.260686	6	2.002348	-0.587153	0.289954
6	0.694521	-1.059413	0.088225	6	0.698892	-1.060022	0.084361	6	0.694521	-1.059413	0.088225
6	0.469079	-2.439616	-0.060975	6	0.470454	-2.450269	-0.050431	6	0.469079	-2.439616	-0.060975
6	-0.878467	-2.92185	-0.31077	6	-0.884741	-2.933209	-0.287863	6	-0.878467	-2.92185	-0.31077
6	-1.967894	-2.023003	-0.218881	6	-1.967709	-2.02175	-0.210266	6	-1.967894	-2.023003	-0.218881
6	-3.288577	-2.511852	-0.360854	6	-3.287158	-2.52429	-0.33963	6	-3.288577	-2.511852	-0.360854
6	-4.410152	-1.609474	-0.161803	6	-4.415379	-1.618402	-0.154433	6	-4.410152	-1.609474	-0.161803
6	-4.172736	-0.230487	-0.017748	6	-4.176675	-0.22935	-0.0232	6	-4.172736	-0.230487	-0.017748

6	-5.258325	0.649448	0.130072	6	-5.269174	0.652237	0.098405	6	-5.258325	0.649448	0.130072
6	-5.018357	2.067793	0.218603	6	-5.029011	2.072423	0.189331	6	-5.018357	2.067793	0.218603
6	2.687788	3.571217	0.511166	6	2.697379	3.577835	0.514204	6	2.687788	3.571217	0.511166
6	3.745789	2.689656	0.662563	6	3.7591	2.694399	0.631321	6	3.745789	2.689656	0.662563
6	3.528202	1.341911	0.588171	6	3.544089	1.345681	0.540957	6	3.528202	1.341911	0.588171
6	-1.854376	5.789757	-0.127469	6	-1.847729	5.802343	-0.083025	6	-1.854376	5.789757	-0.127469
6	-0.558667	6.253042	-0.205735	6	-0.555063	6.282837	-0.155644	6	-0.558667	6.253042	-0.205735
6	0.505734	5.38696	-0.070513	6	0.501298	5.401361	-0.028845	6	0.505734	5.38696	-0.070513
6	-6.072607	2.977966	0.357864	6	-6.084222	2.985792	0.314567	6	-6.072607	2.977966	0.357864
6	-5.836034	4.323129	0.424711	6	-5.84489	4.330902	0.400388	6	-5.836034	4.323129	0.424711
6	-4.54046	4.803384	0.328763	6	-4.54711	4.812719	0.338276	6	-4.54046	4.803384	0.328763
6	-5.70527	-2.084412	-0.092504	6	-5.715506	-2.090605	-0.095426	6	-5.70527	-2.084412	-0.092504
6	-6.774322	-1.219955	0.077592	6	-6.78564	-1.220425	0.040862	6	-6.774322	-1.219955	0.077592
6	-6.555826	0.126175	0.174511	6	-6.568443	0.128336	0.123998	6	-6.555826	0.126175	0.174511
6	-1.121986	-4.242268	-0.644792	6	-1.131599	-4.260271	-0.599129	6	-1.121986	-4.242268	-0.644792
6	-2.408723	-4.69143	-0.849047	6	-2.41573	-4.727606	-0.796881	6	-2.408723	-4.69143	-0.849047
6	-3.482013	-3.839878	-0.694807	6	-3.480419	-3.859611	-0.65076	6	-3.482013	-3.839878	-0.694807
6	3.051767	-1.509041	0.377999	6	3.066163	-1.511515	0.333533	6	3.051767	-1.509041	0.377999
6	2.818248	-2.852205	0.272299	6	2.829111	-2.856708	0.243585	6	2.818248	-2.852205	0.272299
6	1.531891	-3.315412	0.05148	6	1.538925	-3.32532	0.054829	6	1.531891	-3.315412	0.05148
1	2.885641	4.627561	0.588591	1	2.891057	4.634601	0.605446	1	2.885641	4.627561	0.588591
1	4.741945	3.069282	0.836931	1	4.759345	3.073242	0.795406	1	4.741945	3.069282	0.836931
1	4.370457	0.680623	0.697735	1	4.388838	0.682188	0.626619	1	4.370457	0.680623	0.697735
1	-2.660914	6.49467	-0.239961	1	-2.663278	6.501671	-0.183057	1	-2.660914	6.49467	-0.239961
1	-0.375121	7.305028	-0.371438	1	-0.371538	7.336804	-0.312208	1	-0.375121	7.305028	-0.371438
1	1.504686	5.783741	-0.140272	1	1.506169	5.790022	-0.087339	1	1.504686	5.783741	-0.140272
1	-7.090341	2.633596	0.423297	1	-7.10389	2.640059	0.356861	1	-7.090341	2.633596	0.423297
1	-6.657913	5.012795	0.549977	1	-6.67018	5.020872	0.517923	1	-6.657913	5.012795	0.549977
1	-4.381855	5.866783	0.399122	1	-4.385032	5.875112	0.425067	1	-4.381855	5.866783	0.399122
1	-5.906538	-3.141225	-0.150573	1	-5.914256	-3.149187	-0.141819	1	-5.906538	-3.141225	-0.150573
1	-7.779675	-1.611424	0.130961	1	-7.794385	-1.609915	0.081757	1	-7.779675	-1.611424	0.130961
1	-7.406092	0.775635	0.292451	1	-7.419295	0.782739	0.218418	1	-7.406092	0.775635	0.292451
1	-0.308677	-4.93567	-0.776963	1	-0.310462	-4.95	-0.716577	1	-0.308677	-4.93567	-0.776963
1	-2.578318	-5.721075	-1.129914	1	-2.586124	-5.761107	-1.065049	1	-2.578318	-5.721075	-1.129914
1	-4.472846	-4.225589	-0.866228	1	-4.477821	-4.239193	-0.808974	1	-4.472846	-4.225589	-0.866228
1	4.06322	-1.176322	0.535694	1	4.080637	-1.17472	0.469152	1	4.06322	-1.176322	0.535694
1	3.635431	-3.553374	0.358224	1	3.650263	-3.557319	0.319983	1	3.635431	-3.553374	0.358224
1	1.37603	-4.379503	-0.012463	1	1.379529	-4.390311	0.004084	1	1.37603	-4.379503	-0.012463

Perylene

Neutral				Radical anion				Radical cation			
6	1.341615	1.518939	-2.517769	6	1.343751	1.51946	-2.542786	6	1.34285	1.517805	-2.531436
6	-0.026685	1.775043	-2.672819	6	-0.008097	1.771123	-2.684343	6	-0.006547	1.76987	-2.682061
6	-0.840692	1.820199	-1.585475	6	-0.83816	1.818943	-1.587354	6	-0.823508	1.81518	-1.576614
6	-0.312463	1.610273	-0.295725	6	-0.317695	1.611208	-0.296333	6	-0.296811	1.607394	-0.293904
6	1.067238	1.352184	-0.136368	6	1.078849	1.349999	-0.135032	6	1.085327	1.348824	-0.134282
6	1.903121	1.30751	-1.284733	6	1.917813	1.304217	-1.281194	6	1.91814	1.303389	-1.275192

6	-1.15097	1.656249	0.836228	6	-1.14867	1.656516	0.838656	6	-1.13164	1.654515	0.831596
6	-0.635328	1.453285	2.077153	6	-0.619612	1.450746	2.092628	6	-0.617252	1.451282	2.090805
6	0.732408	1.197752	2.238107	6	0.728617	1.197427	2.262898	6	0.730898	1.198853	2.251706
6	1.589116	1.1432	1.168801	6	1.604412	1.140813	1.168706	6	1.606334	1.141255	1.162962
6	3.342059	1.036705	-1.118702	6	3.326882	1.039766	-1.11853	6	3.325038	1.039733	-1.11276
6	3.028597	0.875468	1.335104	6	3.013778	0.877991	1.331515	6	3.013381	0.878435	1.325475
6	3.864205	0.829253	0.186602	6	3.852596	0.831409	0.185279	6	3.84611	0.832592	0.184522
6	5.243903	0.57114	0.345955	6	5.249152	0.570218	0.346562	6	5.228242	0.574024	0.344137
6	6.081817	0.521786	-0.786295	6	6.079811	0.523201	-0.788592	6	6.062885	0.525904	-0.781457
6	5.565597	0.721459	-2.027512	6	5.5504	0.727098	-2.042723	6	5.548328	0.728126	-2.040754
6	4.198039	0.97801	-2.188376	6	4.202253	0.980849	-2.212941	6	4.200237	0.980881	-2.201626
6	3.590827	0.668145	2.568503	6	3.588246	0.665023	2.593307	6	3.588897	0.665245	2.581825
6	4.959296	0.412998	2.723638	6	4.940177	0.413833	2.734888	6	4.938351	0.413513	2.732465
6	5.772721	0.364547	1.636	6	5.769923	0.364204	1.637739	6	5.755131	0.367238	1.626927
1	1.950779	1.492289	-3.406902	1	1.956103	1.491736	-3.429714	1	1.952099	1.49014	-3.419775
1	-0.4271	1.935967	-3.663988	1	-0.415933	1.932711	-3.675263	1	-0.415275	1.930728	-3.668752
1	-1.898837	2.016831	-1.691833	1	-1.896643	2.016201	-1.700405	1	-1.881028	2.011972	-1.686472
1	-2.205121	1.855043	0.698712	1	-2.204787	1.855382	0.707584	1	-2.186054	1.853094	0.697622
1	-1.27458	1.487403	2.948271	1	-1.265759	1.487053	2.961846	1	-1.263036	1.488181	2.955744
1	1.09912	1.042904	3.240031	1	1.099087	1.042475	3.263438	1	1.098153	1.044899	3.25293
1	7.13593	0.32278	-0.648792	1	7.135919	0.324271	-0.657547	1	7.117288	0.327248	-0.6475
1	6.204288	0.68417	-2.898909	1	6.196195	0.688976	-2.912119	1	6.19395	0.690223	-2.90577
1	3.830832	1.130104	-3.190539	1	3.831432	1.133871	-3.213649	1	3.832802	1.133894	-3.202935
1	2.982151	0.697526	3.457883	1	2.976243	0.694592	3.480418	1	2.979822	0.693836	3.470255
1	5.360269	0.255207	3.715086	1	5.34834	0.254076	3.725965	1	5.347262	0.253627	3.719242
1	6.830897	0.16808	1.742373	1	6.828428	0.167058	1.750805	1	6.812674	0.170548	1.736786

Pyrene

Neutral				Radical anion				Radical cation			
6	-0.365956	1.827635	-4.208667	6	-0.36592	1.808168	-4.195265	6	-0.365959	1.808979	-4.194166
6	-0.365959	0.782991	-3.22395	6	-0.365917	0.779218	-3.237637	6	-0.365969	0.79405	-3.220985
6	-0.365953	3.121816	-3.851159	6	-0.365941	3.131622	-3.829664	6	-0.365939	3.130369	-3.829127
6	-0.365953	1.137855	-1.860852	6	-0.365923	1.137305	-1.861009	6	-0.365954	1.146498	-1.858472
6	-0.365954	3.512751	-2.469873	6	-0.365951	3.523006	-2.479663	6	-0.365945	3.501722	-2.472979
6	-0.365952	2.508422	-1.482257	6	-0.365927	2.508962	-1.482095	6	-0.365964	2.499763	-1.484629
1	-0.365954	1.547736	-5.253713	1	-0.365915	1.536531	-5.244596	1	-0.365961	1.537446	-5.240709
6	-0.365964	-0.561473	-3.574716	6	-0.365931	-0.594004	-3.590544	6	-0.365983	-0.573294	-3.572699
6	-0.365953	0.133524	-0.873238	6	-0.365936	0.123264	-0.863441	6	-0.365935	0.144542	-0.87012
6	-0.365953	2.863285	-0.119159	6	-0.365941	2.867058	-0.105472	6	-0.365972	2.852227	-0.122124
6	-0.36595	-1.200345	-1.262326	6	-0.365999	-1.236363	-1.265449	6	-0.365951	-1.20945	-1.270128
6	-0.365956	-1.542745	-2.601362	6	-0.36599	-1.563882	-2.607207	6	-0.365976	-1.55762	-2.60547
1	-0.365966	-0.835233	-4.621366	1	-0.365931	-0.869659	-4.637568	1	-0.365971	-0.844498	-4.619852
6	-0.365956	0.524457	0.508048	6	-0.365918	0.514655	0.486554	6	-0.365925	0.515907	0.48603
1	-0.365945	-1.97243	-0.504531	1	-0.366046	-2.010245	-0.508284	1	-0.365943	-1.979599	-0.510583
1	-0.365956	-2.584843	-2.889335	1	-0.366043	-2.609396	-2.896164	1	-0.365977	-2.598477	-2.89311
6	-0.365957	4.84662	-2.080786	6	-0.365981	4.882633	-2.077659	6	-0.365937	4.855715	-2.072986
6	-0.365953	4.20775	0.231607	6	-0.365969	4.240282	0.247433	6	-0.365984	4.219575	0.229583

6	-0.365955	5.18902	-0.74175	6	-0.365979	5.210161	-0.735905	6	-0.365967	5.2039	-0.737649
1	-0.365948	3.898487	-4.604325	1	-0.365961	3.903413	-4.590737	1	-0.365923	3.900673	-4.587828
6	-0.365959	1.818637	0.865557	6	-0.365916	1.838108	0.852149	6	-0.365948	1.837302	0.851054
1	-0.36596	-0.252215	1.261213	1	-0.365918	-0.257122	1.24764	1	-0.365915	-0.2544	1.244751
1	-0.365956	4.481513	1.278257	1	-0.365977	4.51594	1.294455	1	-0.366007	4.490783	1.27674
1	-0.36596	6.231118	-0.453776	1	-0.366004	6.255684	-0.446964	1	-0.365975	6.244761	-0.450017
1	-0.365963	2.098538	1.910604	1	-0.365904	2.109732	1.901485	1	-0.365947	2.108852	1.897586
1	-0.365959	5.618703	-2.838583	1	-0.366013	5.6565	-2.834833	1	-0.365923	5.625845	-2.832552

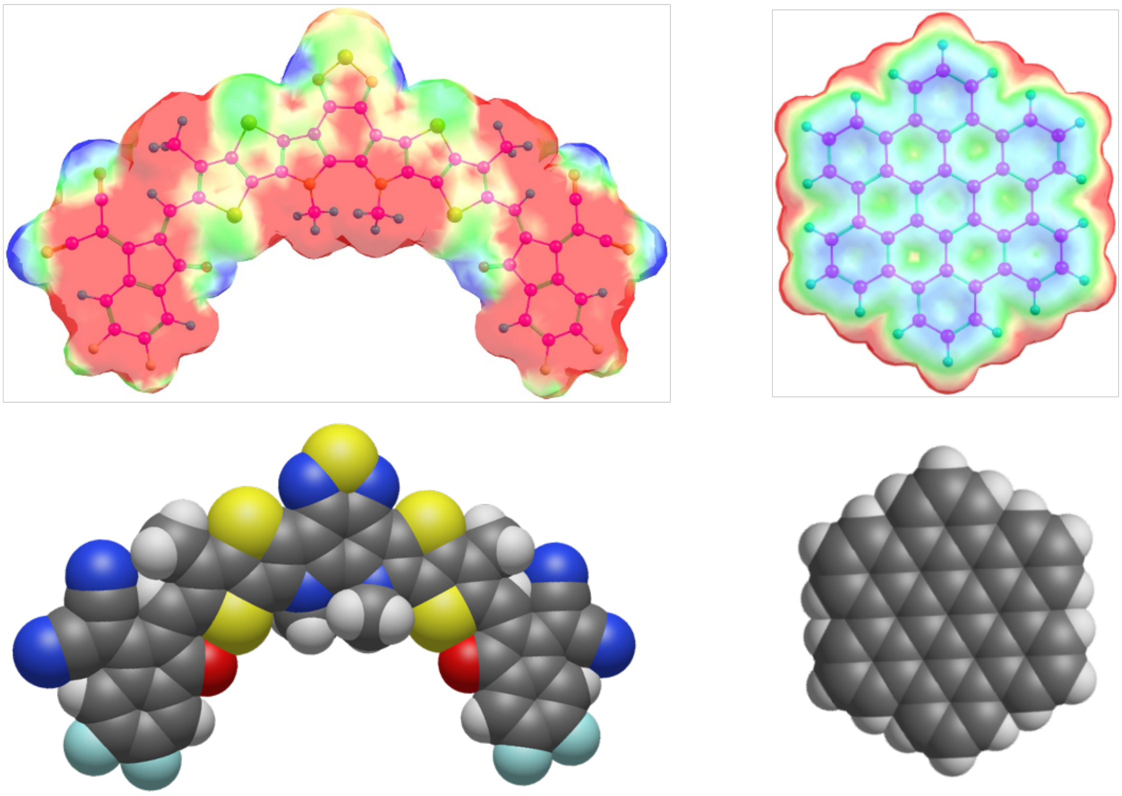

Figure S17: Electrostatic potential (top) and van der Waals structures (bottom) of truncated Y6 and HBC. The values are within the range –0.025 to 0.025. The blue color at indicates the electron rich regions, while the electron-deficient regions are indicated by the red color.

References

(1) Neese, F. Software Update: The ORCA Program System—Version 6.0. *WIREs Comput. Mol. Sci.* **2025**, *15* (2), e70019. https://doi.org/10.1002/wcms.70019.

(2) Neese, F. The SHARK Integral Generation and Digestion System. *J. Comput. Chem.* **2023**, *44* (3), 381–396. https://doi.org/10.1002/jcc.26942.

(3) Müller, M.; Hansen, A.; Grimme, S. *ω* B97X-3c: A Composite Range-Separated Hybrid DFT Method with a Molecule-Optimized Polarized Valence Double- *ζ* Basis Set. *J. Chem. Phys.* **2023**, *158* (1), 014103. https://doi.org/10.1063/5.0133026.

(4) Wittmann, L.; Gordiy, I.; Friede, M.; Helmich-Paris, B.; Grimme, S.; Hansen, A.; Bursch, M. Extension of the D3 and D4 London Dispersion Corrections to the Full Actinides Series. *Phys. Chem. Chem. Phys.* **2024**, *26* (32), 21379–21394. https://doi.org/10.1039/D4CP01514B.

(5) Gerase, Y. T.; Elmanova, A.; Finkelmeyer, S. J.; Dellith, A.; Dellith, J.; Guthmuller, J.; Ryabchykov, O.; Bocklitz, T; Fabozzi, F. G.; Severin, N.; Hecht, S.; Renn, L.; Borchert, J.; Weitz, R. T.; Müller, J.; Koch, C. T.; Presselt, M. Quasi-Two-Dimensional Morphologies of a Non-Fullerene Acceptor Y6 by Interfacial Assembly via Langmuir–Schaefer Technique. *Adv. Mater.* **2025**. DOI:10.1002/adma.202509825.

(6) Hanwell, M. D.; Curtis, D. E.; Lonie, D. C.; Vandermeersch, T.; Zurek, E.; Hutchison, G. R. Avogadro: An Advanced Semantic Chemical Editor, Visualization, and Analysis Platform. *J. Cheminformatics* **2012**, *4* (1), 17. https://doi.org/10.1186/1758-2946-4-17.

(7) Chemcraft - graphical software for visualization of quantum chemistry computations. Version 1.8, build 682. https://www.chemcraftprog.com
