# Supplementary material for: Engineering Interfacial Donor–Acceptor Molecular Cocrystals
Source: J Phys Chem Lett. 2026 Jan 29;17(7):1972–7. doi: 10.1021/acs.jpclett.5c03865 (PMC12927019; doi:10.1021/acs.jpclett.5c03865)
Supplement: Supplementary file 2 [file jz5c03865_si_003.pdf]

jz-2025-038655.R1

Name: Peer Review Information for "Engineering Interfacial Donor–Acceptor Molecular Cocrystals"

First Round of Reviewer Comments

Reviewer: 1

Comments to the Author

The work "Engineering Interfacial Donor–Acceptor Molecular Cocrystals" by N. Severin and colleagues presents a thorough and technically rigorous STM investigation of the interfacial self-assembly and cocrystallization behavior on highly oriented pyrolytic graphite of the non-fullerene acceptor Y6 with a series of polycyclic aromatic hydrocarbons. The work provides a coherent structural picture of donor–acceptor cocrystals at the solid–liquid interface, supported by high-quality STM data and complemented by DFT calculations that rationalize the dominant intermolecular interactions. The results are novel and of clear interest to the communities working on organic semiconductors, supramolecular assembly, and interfacial nanostructures. Without criticizing the quality of the report, I believe that its scope and impact appear to align more with a comprehensive methodology and structural contribution than with the level of immediacy and urgency typically expected for publication in the Journal of Physical Chemistry Letters. In my professional assessment, this manuscript would be better suited for publication in The Journal of Physical Chemistry B. In this journal, its detailed analysis and systematic exploration of polymorphism, solvent effects, and cocrystallization phenomena would reach the appropriate audience and receive proper contextualization.

Please note the following minor aspects:

The work of Ling Yang and col., "Molecular-Level Resolution of the Crucial Differences in the Morphologies of Non-Fullerene Acceptor Y Derivatives/Donor Polymer PM6 Heterogeneous Films" (Surfaces and Interfaces, 55, 2024, 105418,

https://doi.org/10.1016/j.surfin.2024.105418), should be analyzed and commented on in the MS. The paper describes the morphologies of Y5, Y6, and Y7/PM6 heterogeneous films with molecular resolution through scanning tunneling microscopy and analyzes them through DFT calculations.

Additionally, the work "Photovoltaic Performance and Scanning Tunneling Microscopy Analysis of PM6:Y7-Based Organic Solar Cells" by Andrés Plaza-Martínez (Sustainable Energy Fuels, 2025, 9, 2678, DOI: 10.1039/D5SE00278H) is a direct antecedent of the MS. In this work, the nanomorphology of PM6, Y7, and PM6:Y7 monolayers was analyzed by STM under the liquid/solid interface approach.

Please review Line 16 on page 2 in the first column. The tita unit is incorrect.

On page 3, line 1, in the second column, I believe the authors should mention Figure 4a instead of 2a.

Reviewer: 2

Comments to the Author

This manuscript describes the formation of molecular self-assemblies of the non-fullerene acceptor Y6 on graphite surfaces. The study is based on STM imaging at the solid-liquid interface. The authors provide extensive characterization of the crystallization behaviour of Y6 in the absence and presence of polycyclic aromatic hydrocarbons which serve as donor component in two-dimensional cocrystals. The formation of the observed multicomponent supramolecular structures is interpreted in terms of dipole-dipole and pi-pi stacking interactions, as supported by DFT calculations.

Overall the manuscript contains original results, suitable for publication in JPCL. I recommend acceptance subject to minor revision, as follows:

1) The surface packing density is calculated as the number of molecules per unit cell area. The authors should add 1-2 sentences in the Supporting Information, explaining how they actually counted molecules.

2) The authors show an interesting case of assembly obtained by changing the concentration of the Y6 solution. It would be interesting to understand why stable assemblies are only formed within a specific range of concentration.

Author's Response to Peer Review Comments:

see uploaded cover letter as well as uploaded point-by-point reply

**Point-by-point reply to referees' and editor's comments and changes for jz-2025-038655:**

# Reviewer: 1

**Comments:** The work "Engineering Interfacial Donor–Acceptor Molecular Cocrystals" by N. Severin and colleagues presents a thorough and technically rigorous STM investigation of the interfacial self-assembly and cocrystallization behavior on highly oriented pyrolytic graphite of the non-fullerene acceptor Y6 with a series of polycyclic aromatic hydrocarbons. The work provides a coherent structural picture of donor–acceptor cocrystals at the solid–liquid interface, supported by high-quality STM data and complemented by DFT calculations that rationalize the dominant intermolecular interactions. The results are novel and of clear interest to the communities working on organic semiconductors, supramolecular assembly, and interfacial nanostructures. Without criticizing the quality of the report, I believe that its scope and impact appear to align more with a comprehensive methodology and structural contribution than with the level of immediacy and urgency typically expected for publication in the Journal of Physical Chemistry Letters. In my professional assessment, this manuscript would be better suited for publication in The Journal of Physical Chemistry B. In this journal, its detailed analysis and systematic exploration of polymorphism, solvent effects, and cocrystallization phenomena would reach the appropriate audience and receive proper contextualization.

***Reply**: We thank the reviewer for the positive comments concerning the quality of our work. Considering the actual importance of Y6 across supramolecular chemistry and its wide application in organic (opto)electronic devices, we are confident that our manuscript showcases a clear and concise advance in the field and therefore perfectly suits the scope of JPC Letters – supported as well by Reviewer 2.*

1. The work of Ling Yang and col., "Molecular-Level Resolution of the Crucial Differences in the Morphologies of Non-Fullerene Acceptor Y Derivatives/Donor Polymer PM6 Heterogeneous Films" (Surfaces and Interfaces, 55, 2024, 105418, https://doi.org/10.1016/j.surfin.2024.105418), should be analyzed and commented on in the MS. The paper describes the morphologies of Y5, Y6, and Y7/PM6 heterogeneous films with molecular resolution through scanning tunneling microscopy and analyzes them through DFT calculations. Additionally, the work "Photovoltaic Performance and Scanning Tunneling Microscopy Analysis of PM6:Y7-Based Organic Solar Cells" by Andrés Plaza-Martínez (Sustainable Energy Fuels, 2025, 9, 2678, DOI: 10.1039/D5SE00278H) is a direct antecedent of the MS. In this work, the nanomorphology of PM6, Y7, and PM6:Y7 monolayers was analyzed by STM under the liquid/solid interface approach.

***Reply & Action**: The work "Molecular-Level Resolution of the Crucial Differences in the Morphologies of NonFullerene Acceptor Y Derivatives/Donor Polymer PM6 Heterogeneous Films (Surfaces and Interfaces, 55, 2024, 105418, https://doi.org/10.1016/j.surfin.2024.105418)" by Yang et al. represents the initial report on the selfassembly of Yn derivatives (n = 5-7) and their heterogeneous films with PM6 as analyzed by STM at the solidliquid interface. While the authors include many STM images of Y6 in the report, they did not report the formation of two distinct*

*polymorphs, nor did they observe long range interpenetration of the donor–acceptor systems on graphite when blended with the donor polymer PM6. Furthermore, no experimental unit cell parameters for the supramolecular structures have been reported in the manuscript. Since this study in principle represents the first report of Y6 supramolecular assemblies, we have of course cited it – yet due to the work's important limitations the corresponding sentence in our introductory section reads: "… Yet, understanding and controlling Y6 interfacial crystallization behavior as well as preventing phase separation at the interface remain key challenges for the design of D–A systems.[reference to Yang et al.]".*

*Regarding the work "Performance and Scanning Tunneling Microscopy Analysis of PM6:Y7-Based Organic Solar Cells" by Andrés Plaza-Martínez (Sustainable Energy Fuels, 2025, 9, 2678, DOI: 10.1039/D5SE00278H), the authors report on an organic solar cell with high PCE by blending Y7 with PM6. Although they could not structurally resolve and assign the supramolecular organization of Y7 or PM6:Y7, we agree with the reviewer about the significance of this work and have now included it as new reference 20 in our manuscript.*

2. Please review Line 16 on page 2 in the first column. The tita unit is incorrect.

***Reply & Action:*** *We thank the reviewer for noticing the typo in our text. We now removed "nm" after the angle "θ" value.*

3. On page 3, line 1, in the second column, I believe the authors should mention Figure 4a instead of 2a.

***Reply & Action:*** *We thank the Reviewer for the comment and apologize for the confusion. In fact, in the sentence on Page 3, Line 1, we are referring to the nanopores (or voids) that are present within the pristine self-assembly of Y6 on HOPG and that could be used to accommodate HBC molecules. These voids are indeed visible in the STM image showed in Figure 2a, as referred by us in the main text. However, we have now rephrased the initial sentence in order to avoid misunderstanding: "As the main polymorph of Y6 at the HOPG/1-PO interface presents defined nanopores (voids visible in the STM image of Figure 2a) …"*

# Reviewer: 2

**Comments:** This manuscript describes the formation of molecular self-assemblies of the non-fullerene acceptor Y6 on graphite surfaces. The study is based on STM imaging at the solid-liquid interface. The authors provide extensive characterization of the crystallization behaviour of Y6 in the absence and presence of polycyclic aromatic hydrocarbons which serve as donor component in two-dimensional cocrystals. The formation of the observed multicomponent supramolecular structures is interpreted in terms of dipole-dipole and pi-pi stacking interactions, as supported by DFT calculations. Overall the manuscript contains original results, suitable for publication in JPCL. I recommend acceptance subject to minor revision

***Reply*****:** *We appreciate the reviewer´s clear analysis of our work and are thankful for the positive comments.*

1. The surface packing density is calculated as the number of molecules per unit cell area. The authors should add 1-2 sentences in the Supporting Information, explaining how they actually counted molecules.

***Reply & Action*****:** *The surface packing density was obtained by counting the number of molecules within a single experimentally determined unit cell in the STM images. Molecules that lie partially inside the unit cell were counted fractionally according to their area within the unit cell (as example, two half-occupied molecules were counted as one whole molecule).*

*We will add the following sentence to the Materials and Method section in the SI file: “$\rho_S$ was obtained by counting the number of molecules within a single experimentally determined unit cell in the STM image. Molecules that are partially overlapping the unit cell boundary were counted fractionally according to their area within the unit cell”*

2. The authors show an interesting case of assembly obtained by changing the concentration of the Y6 solution. It would be interesting to understand why stable assemblies are only formed within a specific range of concentration.

***Reply***: *We agree and share with the reviewer the interest on the understanding of the crystallization behavior of Y6 on surfaces. As revealed by the STM images showed in Figure 3b, Y6 forms stable assemblies only within a narrow range of concentration. No ordered supramolecular structure could be observed either at lower or higher concentration (Figure 3a and Figure 3c, respectively). This effect could be attributed to a classical (bulk) crystallization behavior: when the concentration of Y6 is too low, there are no enough molecules on the surface to nucleate and stabilize self-assembly domains via intermolecular interactions, so most probably at this stage only isolated molecules or small clusters are present. In contrast, too high concentration of Y6 on HOPG might lead to rapid nucleation, kinetic aggregation, and multilayering processes, giving rise to amorphous (or less ordered) structures.*

## Manuscript Formatting Request by the Editor

1. Author names and affiliations should be present at the beginning of the manuscript underneath the title. Each affiliation should have a label and the label should be present by the associated author name. Please see the following Author and Address Information sheet for examples: https://pubsapp.acs.org/paragonplus/submission/author-address-information.pdf.

***Action***: *Done.*

2. Please remove the section headings from your manuscript file, e.g., Introduction, Results and Discussion, Conclusion. Experimental section headings as well as paragraph headings are okay.

***Action***: *Done.*

3. The TOC graphic should fit in an area no larger than 3.25 in. × 1.75 in. (approx. 8.25 cm × 4.45 cm) and should have adequate resolution and clarity. Confirm that all text is legible at this size.

***Action***: *We now updated the TOC graphic – the text is fully readable and the graphic has the adequate resolution.*

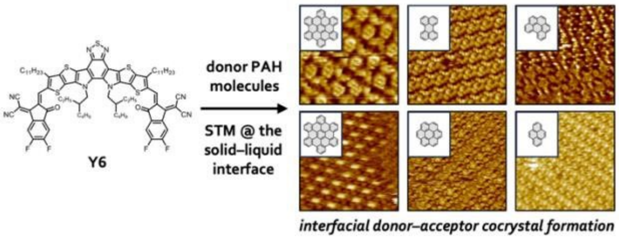


Updated TOC graphic

jz-2025-038655.R2

Name: Peer Review Information for "Engineering Interfacial Donor–Acceptor Molecular Cocrystals"

Second Round of Reviewer Comments

Reviewer: 1

Comments to the Author

The authors addressed all comments in a satisfactory manner.

While I believe that the work meets the necessary standards, its publication is not a matter of urgency. I recommend that the editor make a final decision based on the comments made by the other reviewers who analyzed the work.

Author's Response to Peer Review Comments:

**Prof. Stefan Hecht, Ph.D.**
Einstein Professor
Organic Chemistry & Functional Materials
Department of Humboldt-Universität zu BerlinChemistry
Brook12489 Berlin-Taylor, Germany-Str. 2
Center for the Science of Materials Berlin
Humboldt-Universität zu Berlin
Zum Großen Windkanal 2 12489 Berlin, Germany sh@hu-berlin.de www.hechtlab.de

Senior Editor

*The Journal of Physical Chemistry Letters*

January 22, 2026

**Final manuscript jz-2025-038655**

HUMBOLDT-UNIVERSITÄT ZU BERLIN

Dear Editor:

Thank you very much for in principle accepting our manuscript. We have made the final adjustments as requested by your editorial office, i.e. inserted the emails of the corresponding authors on the first page and removed all headings throughout the main text.

We are looking forward to see our work published in a forthcoming issue of *The Journal of Physical Chemistry Letters*.

With kind regards on behalf of all authors,

Stefan Hecht
